# The Vital Role of Blood Flow-Induced Proliferation and Migration in Capillary Network Formation in a Multiscale Model of Angiogenesis

**DOI:** 10.1371/journal.pone.0128878

**Published:** 2015-06-05

**Authors:** Hossein Bazmara, Madjid Soltani, Mostafa Sefidgar, Majid Bazargan, Mojtaba Mousavi Naeenian, Arman Rahmim

**Affiliations:** 1 Department of Mechanical Engineering, K. N. T. University of Technology, Tehran, Iran; 2 Division of Nuclear Medicine, Department of Radiology and Radiological Science, Johns Hopkins University, School of Medicine, Baltimore, MD, United States of America; University Francisco de Vitoria School of Medicine, SPAIN

## Abstract

Sprouting angiogenesis and capillary network formation are tissue scale phenomena. There are also sub-scale phenomena involved in angiogenesis including at the cellular and intracellular (molecular) scales. In this work, a multiscale model of angiogenesis spanning intracellular, cellular, and tissue scales is developed in detail. The key events that are considered at the tissue scale are formation of closed flow path (that is called loop in this article) and blood flow initiation in the loop. At the cellular scale, growth, migration, and anastomosis of endothelial cells (ECs) are important. At the intracellular scale, cell phenotype determination as well as alteration due to blood flow is included, having pivotal roles in the model. The main feature of the model is to obtain the physical behavior of a closed loop at the tissue scale, relying on the events at the cellular and intracellular scales, and not by imposing physical behavior upon it. Results show that, when blood flow is considered in the loop, the anastomosed sprouts stabilize and elongate. By contrast, when the loop is modeled without consideration of blood flow, the loop collapses. The results obtained in this work show that proper determination of EC phenotype is the key for its survival.

## Introduction

Angiogenesis is the main process involved in tumor growth and cancer development [1]. In tumor-induced angiogenesis, pre-existing vessels form new vessels toward the tumor. The induction of the nearby vessels to build new vessels is accomplished via release of tumor angiogenic factors (TAFs). TAFs diffuse in the surrounding tissues and reach nearby vessels. ECs of the nearby vessels start to migrate up along the TAFs gradient. Newly formed sprouts extend toward the tumor. During the extension, sprouts may fuse together and form closed flow pathway, which is called in this article as a closed loop or simply a loop. Blood flow starts in the loop and induces the ECs to grow and migrate. In vivo experiments on rabbit cornea [2] and mouse eye [3] show that the loops elongate following formation. Loops fuse with other loops or sprouts, and construct a network of capillaries. In fact, the capillary network is a collection of loops with different sizes, which circulates the blood in the area that is closer to or within a tumor. Though dozens of cells and molecules are involved, ECs play the main role during the entire process of angiogenesis [4].

Angiogenesis covers multiple biological scales including molecular, cellular, tissue, and organ scales [5]. Multiple mathematical models of angiogenesis have been presented; however, the models usually do not cover all the scales and limited events in each scale are considered [5].

In angiogenesis, key events such as branching, capillary network formation, blood flow, and vessel adaption are at the tissue scale[5]. Since most experimental observations are at the tissue scale, this scale is the most interesting one for modeling purposes. Anderson et al. [6] presented a hybrid discrete-continuum model of tumor-induced angiogenesis. The authors also observed blood flow in the capillary network and effect of blood flow in vascular remodeling [7]. Cai et al. [8] developed a coupled model of tumor angiogenesis, tumor growth and blood perfusion. Milde et al. [9] also proposed a 3D model of sprouting angiogenesis.

Detailed investigation of cellular and molecular scales within the model enables more faithful reproduction of the real physics of the phenomenon. This is the rational to add cellular and molecular scales to the models, which construct multiscale models. Owen et al. [10] developed a multiscale model that combines blood flow, angiogenesis, and vascular remodeling. This model also considers subcellular scales. To investigate tumor's growth dynamics, they developed this model to include nutrient and growth factor transport, movement and interactions of normal and tumor cells, and nutrient-dependent cell cycle dynamics [11]. Qutub et al. [5] developed a multiscale model of angiogenesis and presented a map to classify the different models of angiogenesis and the scales each model covers. Cell based models have shown excellent ability in modeling growth and migration of ECs [12–15]. Bauer et al. [12,13] presented a multiscale model of sprouting angiogenesis, which includes intracellular, cellular, and extracellular scales. The main feature of this model is the ability to predict cell phenotype regarding the extracellular matrix (ECM) structure and environmental conditions. Due to its detailed investigation at the intracellular scale [16,17], this model has an outstanding ability to capture the real physics of the problem; however, it did not thoroughly incorporate tissue scale phenomena. The only tissue scale events that are considered in this model are the diffusion of TAFs in the ECM and sprout formation. Shirinifard et al. developed a cell based model for 3D tumor growth and angiogenesis [18]. Merks et al. presented a cell based model for the initial patterning of ECs, which is based on plausible behavior of ECs [19].

In this paper, a multiscale model of sprouting angiogenesis, including molecular (intracellular), cellular, and tissue scales is developed to capture key events in angiogenesis, including formation of a closed loop, blood flow in the loop, and loop survival after blood flow. In the presented model at the intracellular scale, the cell phenotype and cell phenotype alteration due to blood flow are considered. At the cellular scale, growth and migration of ECs, and EC anastomosis are investigated employing a Glazier–Graner–Hogeweg (GGH) model that is also called cellular Pott’s model. In addition, the heterogeneous ECM structure is considered at the cellular scale. At the tissue scale, formation of a closed loop, blood flow in the loop, and stabilization and elongation of the loop are studied. The environmental conditions of ECs are also incorporated within the model at the tissue scale. All the scales are involved simultaneously within the solution. Combining molecular, cellular, and tissue scale dynamics in this work, the model is able to simulate the initial steps of capillary network formation and effect of blood flow in the capillaries fate.

## Material and Methods

**Fig 1:**
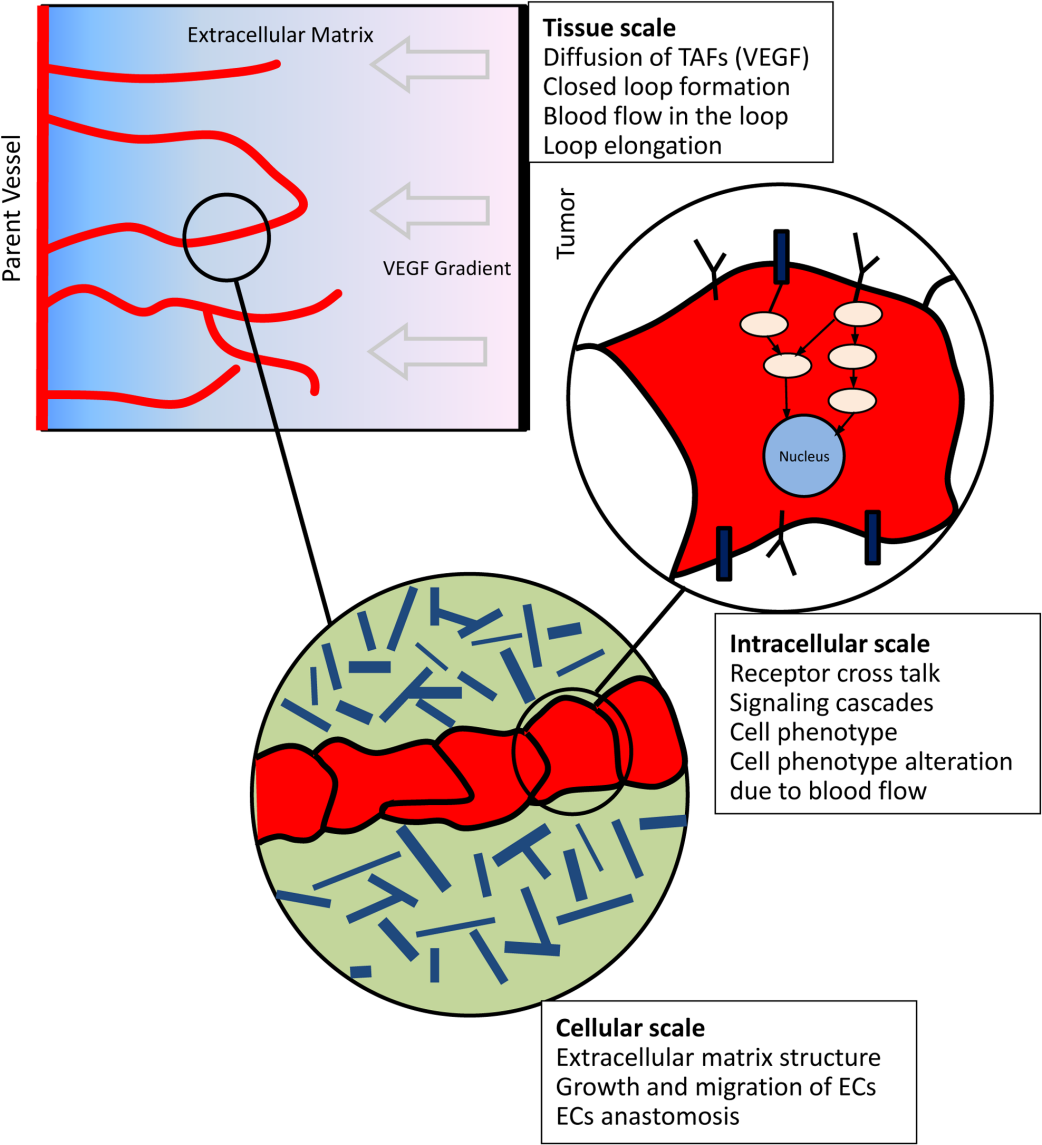
A schematic of the scales involved in the model and the important events considered in each scale. At the tissue scale, single sprouts extend toward the tumor. The sprouts may fuse together and form closed loops. Following anastomosis and formation of a closed loop, blood flow starts in the capillaries. When single sprouts extend, environmental conditions of ECs activate a signaling cascade inside ECs. When blood flow starts, a different signaling cascade is activated inside ECs and consequently, a different behavior is observed from ECs before and after blood flow.

The model presented in this work covers intracellular, cellular, and tissue scales. Each scale is described in a separate section. A schematic of the important events in each scale is presented in Fig 1.

**Fig 2:**
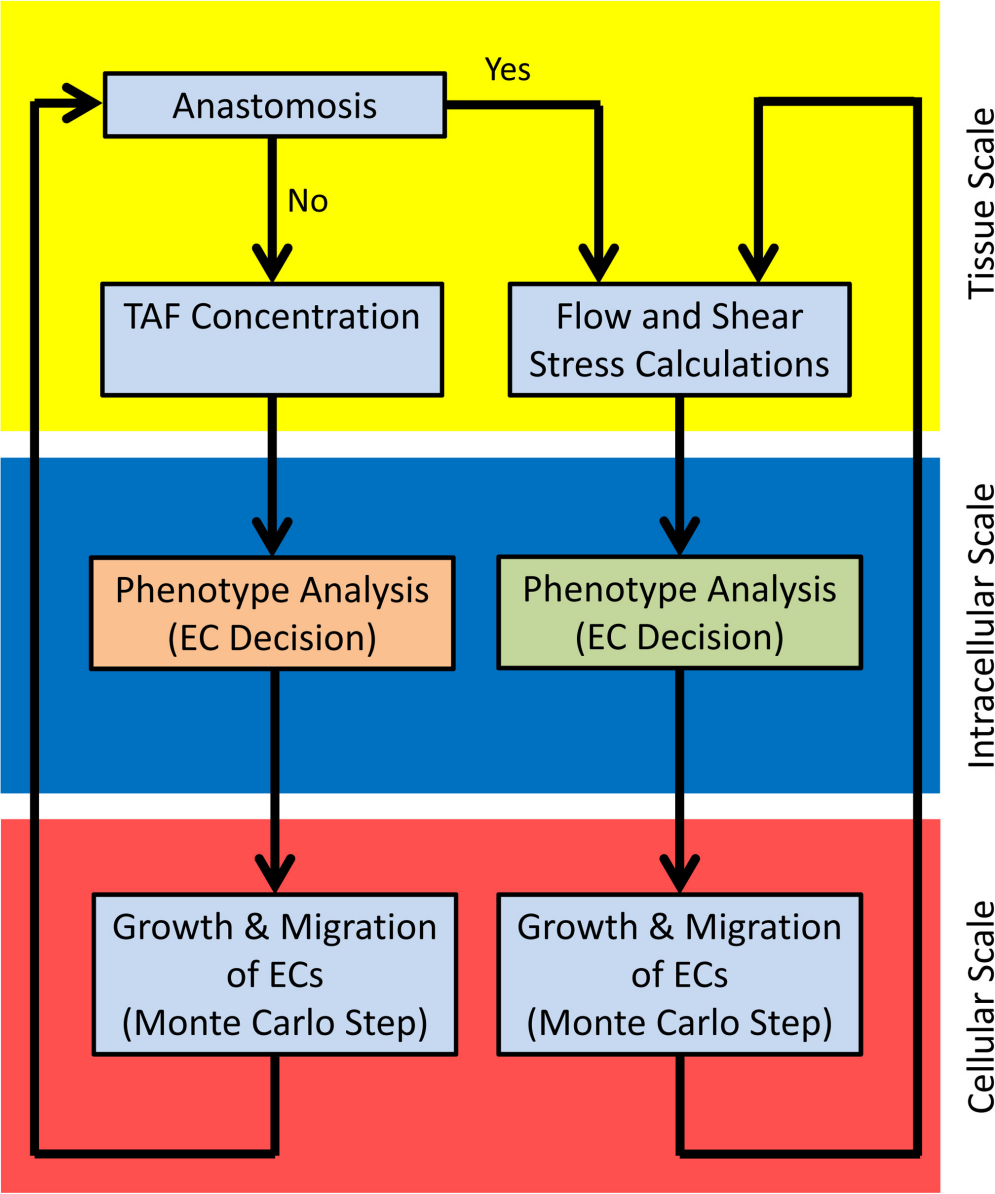
The solution procedure for the multiscale model. The yellow zone represents the tissue scale, the blue zone is intracellular (molecular) scale, and the red zone is cellular scale. In intracellular scale, different signaling cascades are used for EC phenotype determination before and after anastomosis. In cellular scale, growth and migration of ECs are calculated with similar formulation before and after anastomosis; however, the constants in the formulations depend on the predicted phenotype.

The solution procedure and connection between scales are shown in Fig 2.

### Intracellular (molecular) scale

At the intracellular scale, interaction of signaling molecules in the signaling cascades determines cell phenotype, which is the main regulator of EC behavior [20]. Bauer et al. [16,17] developed a Boolean network model for receptor cross talk and intracellular signaling molecules interaction in angiogenesis. The authors also presented a map for EC phenotype based on the environmental signals. The usage of the presented map, however, was limited to ECs in single sprouts without blood flow. In the present work, cell phenotype determination is divided into two separate stages. In the first stage, when sprouts have not yet formed a closed loop and ECs do not sense shear stress of the blood flow, EC phenotype is determined based on the map presented by Bauer et al. [16,17]. This stage is shown by an orange box in intracellular scale in Fig 2. In the second stage, following formation of a closed loop and shear stress activation of ECs, ECs regulate their phenotype in response to shear stress. This stage is shown by a green box in intracellular scale in Fig 2 and is investigated in this section.

The inner layer of blood vessels and capillaries are covered by ECs. ECs experience three main hemodynamic forces in capillaries. The first one is the normal force of pressure due to hydrostatic pressure of blood in the circulatory system. The second force is the tensional force that is exerted in EC longitudinal direction and is created because of diameter changes. The third and most important hemodynamic force is shear stress that is created due to frictional resistance of capillaries against blood flow [21].

ECs sense the shear stress generated by blood flow in capillary walls and transmit the signals to the cell inside, which finally generates a cell response. Shear stress plays an important role in angiogenesis and capillary network hemostasis. Mechanosensing and mechanotransduction are performed by specific proteins on the EC surface [22]. A number of membrane molecules and microdomains mediate mechanotransduction of shear stress and its conversion into intracellular biochemical signals. There are several candidates as shear stress sensors including ion channels, caveolae, G-protein-coupled receptors, tyrosine kinase receptors especially VEGFR2, integrins, glycocalyx, and primary cilia [22–25]. There are also multiple pathways involved in shear stress signal transduction; however, it is not clear exactly which pathways are primary [24]. Though shear stress mechanotransduction has been the subject of many studies, too many aspects remain unclear [24,26].

There is considerable evidence on the effect of shear stress on EC function and phenotype. Cultured ECs reorient their longitudinal axis according to the streamlines of the flow. This will reduce the effective shear stress on ECs [21]. Several studies show that shear stress has a pivotal role on EC survival and prevention of apoptosis [27,28]. There is also evidence that shear stress impacts EC proliferation [25,29]. In wound healing, laminar shear stress enhances EC migration [30,31]. In microcirculation, e.g. for a capillary network, shear stress may play a role in guidance of EC migration along the interstitial flow paths [32]. It is also reported that shear stress stimulates ECs to produce vasodilators [33,34].

At the intracellular scale, experimental studies determine the role of cell surface receptor and intracellular signaling molecules in signaling cascade of shear stress. Integrin is involved in shear stress mechanotransduction [35] and activation of receptor tyrosine kinases (RTKs) [36]. Activation of integrin activates FAK, paxillin, c-Src, Fyn, and P130, which leads to activation of Ras-ERK pathway [37,38]. The ERK pathway is involved in cell growth and proliferation [39]. Shear stress also activates RTKs including VEGFR2 and Tie2. The activation of RTKs is independent from VEGF or angiopoietin [40–43]. Activation of RTKs activates MAPK pathways including ERK, JNK, PI3K, and Akt through activation of Ras. These pathways are the main regulator for cell survival and inhibition of apoptosis [24,25]. Moreover, shear stress causes rapid tyrosine phosphorylation of PECAM-1 [44]. Activation of ERK is dependent on PECAM-1 [45]. PECAM-1, VEGFR2, and VE-cadherin form a complex mechanosensory system. This system has a critical role in transduction of shear stress signals [46]. PECAM-1 and VE-cadherin are necessary for shear stress activation of integrin [23]. After formation of a closed loop and start of blood flow, the flow activates ECs surface receptors. Therefore, when flow is considered in the loop, phosphorylation of RTK is assumed ligand-independent, though VEGF may also contribute in RTK phosphorylation.

**Fig 3:**
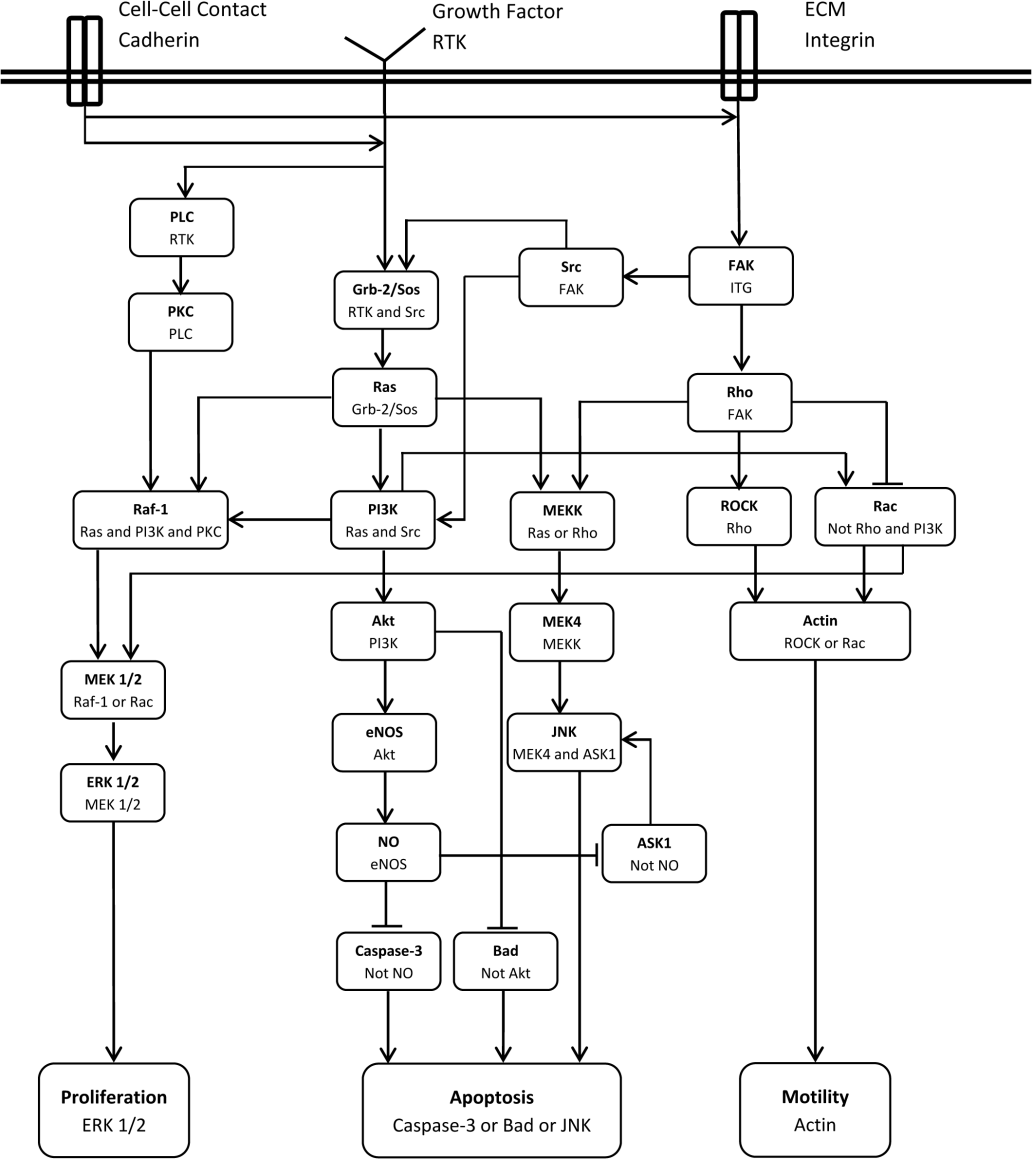
Signaling cascade of shear stress. **An arrow indicates an activation signal while a hammerhead indicates inhibition.** The second line in each box is the combination of input(s) required for activation or inhibition of the molecules.

During angiogenesis key events are regulated by RTKs, integrin, and VE-cadherin [16]. These receptors are considered as the main receptors to develop a signaling cascade for shear stress. To build the signaling cascade of shear stress, multiple observations in the literature about the shear stress activation of ECs is integrated and a signaling cascade for shear stress is proposed here and shown in Fig 3.

The activation or inhibition signals in the cascade are considered as Boolean relations. Table 1 outlines the Boolean dependence relation of the network shown in Fig 3 and the references for each relation.

**Table 1:** Nodes dependence relation and corresponding reference for shear stress signaling cascade.

Node	Dependence Relation	Reference
Integrin	VE-cadherin and Flow	[23,25,37,38]
RTK	Flow	[44]
PLC	RTK	[20,24]
Grb-2/Sos	RTK and Src	[47]
FAK	Integrin	[20,23,40,41]
Src	FAK	[20]
PKC	PLC	[20]
Ras	Grb-2/Sos	[24,40,47]
Rho	FAK	[20]
PI3K	Ras and Src	[16,17]
MEKK	Ras or Rho	[24]
ROCK	Rho	[20]
Rac	Not Rho and PI3K	[16,17]
Raf-1	Ras and PI3K and PKC	[20,21,24,48]
MEK1/2	Raf-1 or Rac	[20,49]
Akt	PI3K	[20,44]
MEK4	MEKK	[24]
Actin	ROCK or Rac	[20]
ERK1/2	MEK1/2	[20,24]
eNOS	Akt	[20,44]
JNK	MEK4 and ASK1	[24,50]
NO	eNOS	[20,44]
ASK1	Not NO	[22]
Caspase-3	Not NO	[50]
Bad	Not Akt	[50]
Proliferation	ERK1/2	[20,51]
Apoptosis	Caspase-3 or Bad or JNK	[50]
Migration	Actin	[20]

A Boolean network model is used to analyze the signaling cascade presented in Fig 3 (see [17], for a detailed description about the Boolean network model and its application in modeling signaling cascades). The results of the model will show the relation between input signals (cell surface receptors status) and cell phenotype.

### Cellular scale

At the cellular scale, the main issue is EC behaviors such as growth, migration, and anastomosis. At this scale, an agent based model is used to simulate EC behavior. A cellular Pott’s model is employed and adapted to show EC growth, migration, apoptosis, and anastomosis. This model is a discretized lattice Monte Carlo model that is developed by Glazier and Graner [52] and is based on the energy minimization principle. The term representing energy in this model is referred to as Hamiltonian. The simulation is started in state A with total energy of E_A_. The model changes from state A to state B and the energy of the system in state B is calculated. If E_B_<E_A_, the change is accepted by the algorithm; otherwise the change is only accepted with a probability according to the Boltzmann distribution.

**Fig 4:**
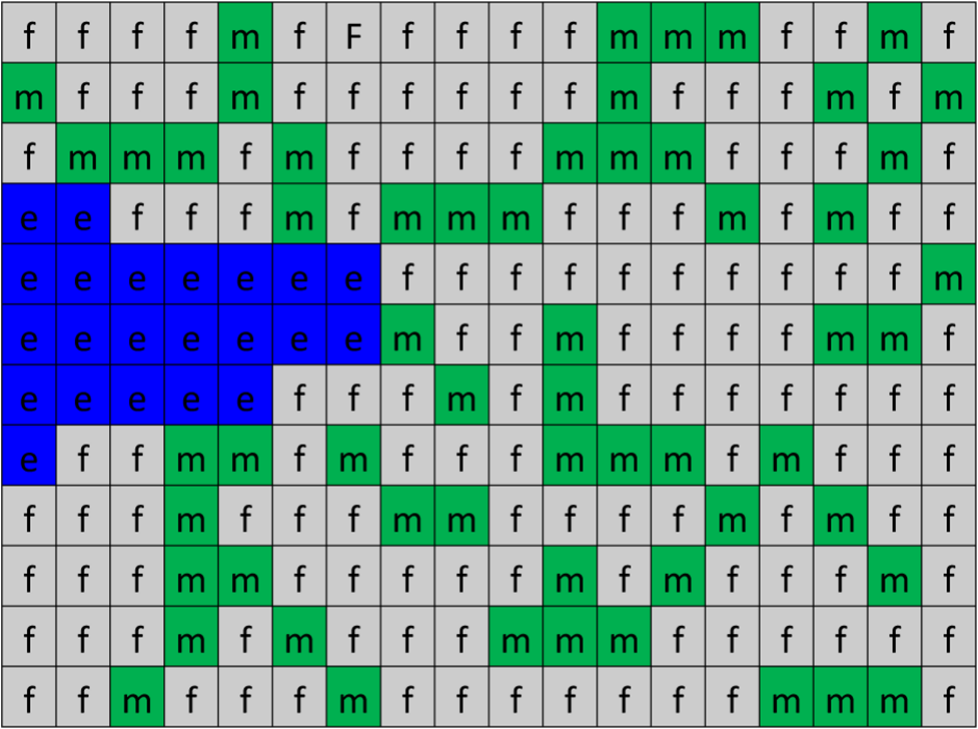
A schematic of the solution domain in Pott’s model at the cellular scale.

To establish the modeling framework, the domain is divided into lattice sites. A schematic of the solution domain is shown in Fig 4. Three entities are defined in this model including matrix fibers, interstitial fluid, and endothelial cells that are shown by m, f, and e, respectively. The lattice sites are filled by the entities, and their evolution through the solution is controlled by Hamiltonian values.

**Fig 5:**
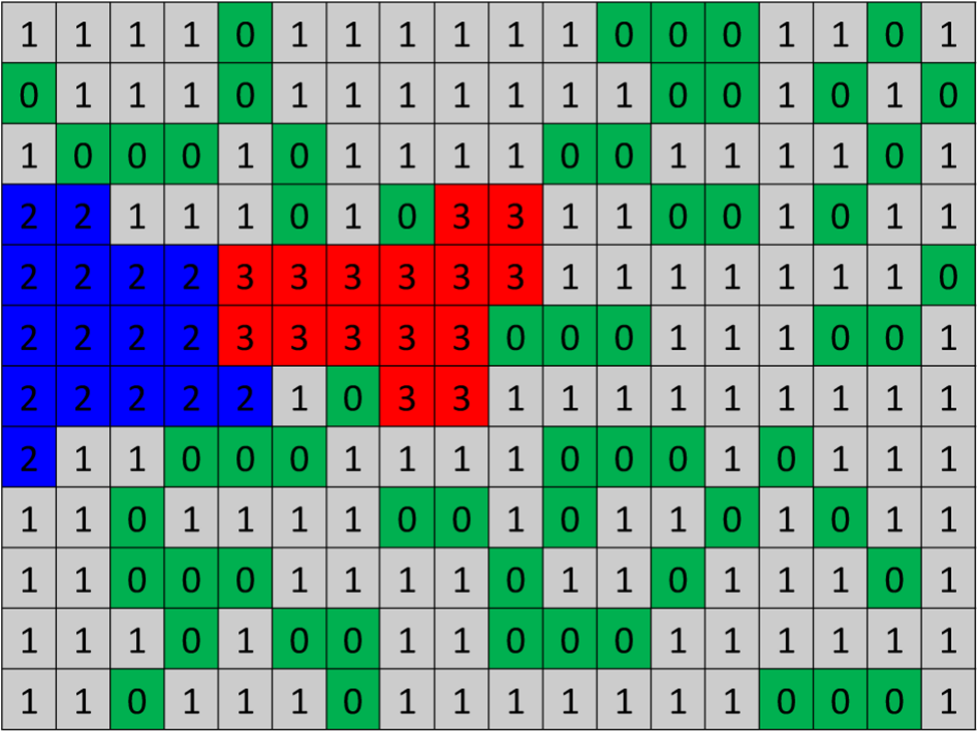
A sample of the solution domain with assigned numbers. 2s and 3s are two ECs, 0s are representing interstitial fluid, and 1s are matrix fibers. Matrix fibers in ECM are distributed randomly.

In the algorithm, numbers are assigned to lattices sites. Since the quantity of ECs is changed during the solution, a number should be assigned to each entity and EC, which is shown by *σ* in the formulation. In this model, 0 is assigned to all lattice sites that are filled by interstitial fluid, 1 is assigned to those that are filled by matrix fibers, and numbers greater than 1, i.e. 2,3,4, … are assigned to ECs. Fig 5 shows a sample of the assigned numbers in the solution domain.

### The extracellular matrix (ECM)

ECM is a composition of interstitial fluid, matrix fibers, and connective tissue cells such as macrophages, fibroblasts, and plasma cells. Interstitial fluid is plasma like fluid, which fills the area between the tissue and vessels. Matrix fibers are composed of collagen and elastin and fill part of ECM. The ratio of matrix fibers volume to ECM volume is defined as matrix density. Using an *in vitro* microfluidic device, Shamloo et al. showed that migration and proliferation of ECs is not possible in very low or high values of matrix densities [53], while intermediate matrix densities enhance migration and proliferation of multiple ECs. Accordingly, a matrix density of 40% is considered in this work. The solution domain is considered to be 240x450 μm. The fibers length is assumed between 5 to 15 μm with 1 μm in diameter. The matrix fibers are distributed randomly (both position and angle) in the domain.

#### Growth and migration of ECs

The physical characteristics and behaviors of ECs are controlled by energy of the system; i.e. the Hamiltonian. Key physical characteristics of ECs are adhesion, growth, and migration.

The first key physical characteristic of an EC is its adhesion to other ECs, or matrix elements. The adhesion energy is computed as follows by

$$E_{adhesion}=\sum_{sites}J_{\tau ,\tau \prime }\left(1-\delta_{\sigma ,\sigma \prime }\right)$$ (1)

J is the adhesion energy between two entities *τ* and *τ*′. *σ* and *σ*′ are numbers of two adjacent lattice sites and *δ* is the Kronecker delta. Kronecker delta has nonzero value only in lattice sites with different numbers in the neighborhood. It should also be mentioned that this values are not calculated in lattice sites that do not have an EC in their neighborhood.

The next key biological characteristic of an EC is its growth and proliferation. ECs proliferate through mitosis. During the cell cycle, ECs grow to double their original volume. The doubled volume is designated as the target volume in the model. When ECs reach to their target volume (twice its initial volume), mitosis starts. After mitosis, the cell is divided into two daughter cells. One cell keeps the original cell ID and the other gets its own unique ID. To show EC growth, a term is added to the Hamiltonian. The initial volume of ECs is considered around 250 *μm* ^2^ [12,54].

$$E_{growth\;constr.}={\sum_{cells}\gamma_{e}\left(a_{\sigma }-A_{\sigma }^{T}\right)}^{2}$$ (2)

*a*_*σ*_ is the current volume of EC and $A_{\sigma }^{T}$ is the target volume of the EC. *γ*_*e*_ is the resistance of EC to the volume change that is interpreted as EC elasticity. When EC volume is less than its target volume, every change in the EC that increases its volume, reduces the total energy of the system and helps to accept the change.

EC migration is the key event in sprouting angiogenesis. Two mechanisms are considered in this model for EC migration: chemotaxis and haptotaxis. Chemotaxis depends on the VEGF gradient in the domain and therefore a simple relation can express its contribution to total energy. Haptotaxis, which is movement of ECs toward adhesion gradients, is naturally modeled in this model via considering different adhesion energies between entities. The chemotactic contribution is

$$E_{Chemotaxis}=\sum_{sites}\chi_{\sigma }\Delta V$$ (3)

where *χ*_*σ*_ is the effective chemical potential and Δ*V* is VEGF gradient. Based on EC phenotype, different effective chemical potentials for ECs in the domain are considered [12].

Another term that should be added is related to cell continuity. An EC is always a continuous medium; however, keeping the continuity of lattice sites of an EC needs a numerical constraint on the model. To keep EC continuity, a term is added to total energy that drastically increases the system energy if the EC loses its continuity [13].

$$E_{continuity}=\sum_{cells}\alpha \left(1-\delta_{a_{\sigma },a_{\sigma }^{\prime }}\right)$$ (4)

*a*_*σ*_ is the cell size and $a_{\sigma }\prime$ is the number of lattice sites that are continuously occupied by the cell σ. α is a large penalty factor to increase system energy in case of difference between *a*_*σ*_ and $a_{\sigma }\prime$.

#### Anastomosis of tip cells

Loop formation is a critical step in sprouting angiogenesis. The loop is formed by fusion of sprouts or anastomosis. Anastomosis is a process in which two tip cells, or a tip cell and a stalk cell, come into contact. When single sprouts extend toward the tumor, they are headed by tip cells, which extend long filopodia. The filopodia is the main sensor for environmental cues and directs the migration of sprout in the nearby tissue. The ability of endothelial tip cells to find each other is quite dependent on filopodial extensions.

Though the morphological events in anastomosis are elucidated in vitro [55] and in vivo [56–59], the cellular and molecular mechanism of the process is yet remaining unclear. There are some plausible explanation about why endothelial tip cells move toward each other, such as VEGF secretion by tip cells [57], EC behavior alteration by ECM components specially fibronectin and laminin [60], and the role of interstitial flow and microvascular pressure [61]; however, there is no definitive description about the mechanism by which two tip cells attract each other. Regardless of the process by which tip cells attract each other, they always find each other and establish filopodial connection; however, not all connections are constructive, i.e. most connections are terminated in short time and no anastomosis occurs.

To implement loop formation in the model, a description of anastomosis at the molecular level is necessary; however, since such a description does not exist, loop formation can be implemented in the model at the cellular level. To add this phenomenon in the cellular level, a term representing attraction of two endothelial tip cells is added to the Hamiltonian:

$$E_{tip\;cell\;attrac.}={\sum_{tip\;cells}\psi \left({\bar{X}}_{1}-{\bar{X}}_{2}\right)}^{2}$$ (5)

where ${\bar{X}}_{1}$ and ${\bar{X}}_{2}$ are centroid of tip cells and ψ is a parameter controlling the attraction.

### Extracellular scale

#### Diffusion of TAFs in ECM

The avascular tumor growth phase cannot continue after the tumor reaches 1–3 mm in diameter [62]. Due to limited diffusion of oxygen, hypoxic regions form in the tumor. The tumor starts to secret TAFs to induce vessels to build new capillaries for it. The main TAF is VEGF that binds to VEGFR2 (the VEGF specific receptor on the EC surface). For a given configuration of parent vessel and tumor, VEGF distribution in the domain has a key role in guidance of sprout migration. VEGF distribution in the domain is governed by a partial differential equation, which considers diffusion, decay, and uptake of VEGF:

$$\frac{\partial V}{\partial t}=D\nabla^{2}V-\lambda V-B(x,y,V)$$ (6)

where V is VEGF concentration, D is diffusion coefficient of VEGF, λ is decay rate of VEGF, and *B* is a function expressing VEGF binding to VEGFR2.

$$B(x,y,V)=\{\begin{array}{c} \beta \;if\;\beta \leq V\;and\;\left\{(x,y)\;\subset endothelial\;cell\right\}\\ V\;if\;0\leq V\;<\beta \;and\;\left\{(x,y)\;\subset endothelial\;cell\right\}\\ 0\;if\;\left\{(x,y)\;⊄endothelial\;cell\right\} \end{array}$$ (7)

Β is the total amount of VEGF that one EC can consume.

The domain size considered in the present study is ∼ 0.4 mm. The avascular tumor size is 1–3 mm, and as such, with a good approximation, it can be assumed that the tumor is a straight line occupying one side of the domain. Assuming a rectangular domain, with a linear tumor in one side and a vessel in the opposite side, the boundary and initial conditions are imposed.

$$\begin{array}{l} V(x,y,0)=0\\ V(0,y,t)=0\;,\;V(L_{1},y,t)=S\;,\;V(x,0,t)=V(x,L_{2},t) \end{array}$$ (8)

The temporal scale of VEGF diffusion and the angiogenesis process are different. VEGF distributes rapidly in the ECM and its concentration can be assumed to be constant during the process. Changes in VEGF concentration is only due to VEGF uptake by ECs and its decay [6,7,63].

#### Loop formation and blood flow in the loop

Sprout formation in angiogenesis and anastomosis of endothelial tip cells create closed loops. After formation of a loop, blood flow starts in the loop. The main effect of blood flow in the capillaries is shear stress.

Laminar flow and pulsatile laminar flow show qualitatively similar effects on ECs [47]. The level of shear stress for human aorta is 10–20 dyne/cm^2^ and for walls of veins is 1–6 dyne/cm^2^ [23]. For single sprouts, wall shear stress is reported to be around 10 dyne/cm^2^ or less [64]. The shear stress has its maximum value in the junction area with parent vessel and decreases along the sprout [64]. This shear is due to blood flow in parent vessel and leakiness of the newly formed capillaries. For capillaries in a network in sprouting angiogenesis, the shear stress is estimated at ∼ 20–40 dyne/cm^2^ [65,66]. ECs respond to shear stress magnitudes as low as 0.1–0.3 dyne/cm^2^ [67], which means that ECs in sprouting angiogenesis are activated by shear stress. It has also been shown that physiological levels of shear stress (∼3 dyne/cm^2^), decrease VEGF-induced activity of ECs [55,61].

In the current study a two-dimensional space is considered for growth and migration of ECs; however, a one-dimensional Poiseuille flow model is employed to calculate shear stress in the capillary walls. Though the ECs grow and migrate in this study in a two-dimensional space, there are three main reasons for using the one-dimensional model for blood flow in the capillary. The first reason is low Reynolds number. The Reynolds number is a non-dimensional number describing the ratio of inertial forces to viscos forces. In blood flow in capillaries, the Reynolds number is very low (less than 1), which means that the flow has a uniform profile and complexities in the flow profile due to geometry of the domain are minimum. The second reason is the viscosity of the blood in capillaries. Pries et al. [68,69] used experimental data to develop formula for the relation between viscosity and vessel diameter. These data are presented in one-dimensional form, so usage of these data in two-dimensional space do not add anything to the model accuracy, even may add some errors due to incompatibility of the viscosity data and space formulation. The third reason is that the exact values of shear stress are not the main concern in this model. In fact, the obtained values for shear stress are just compared with a specified shear stress magnitude to decide about the shear stress activation of ECs.

**Fig 6:**
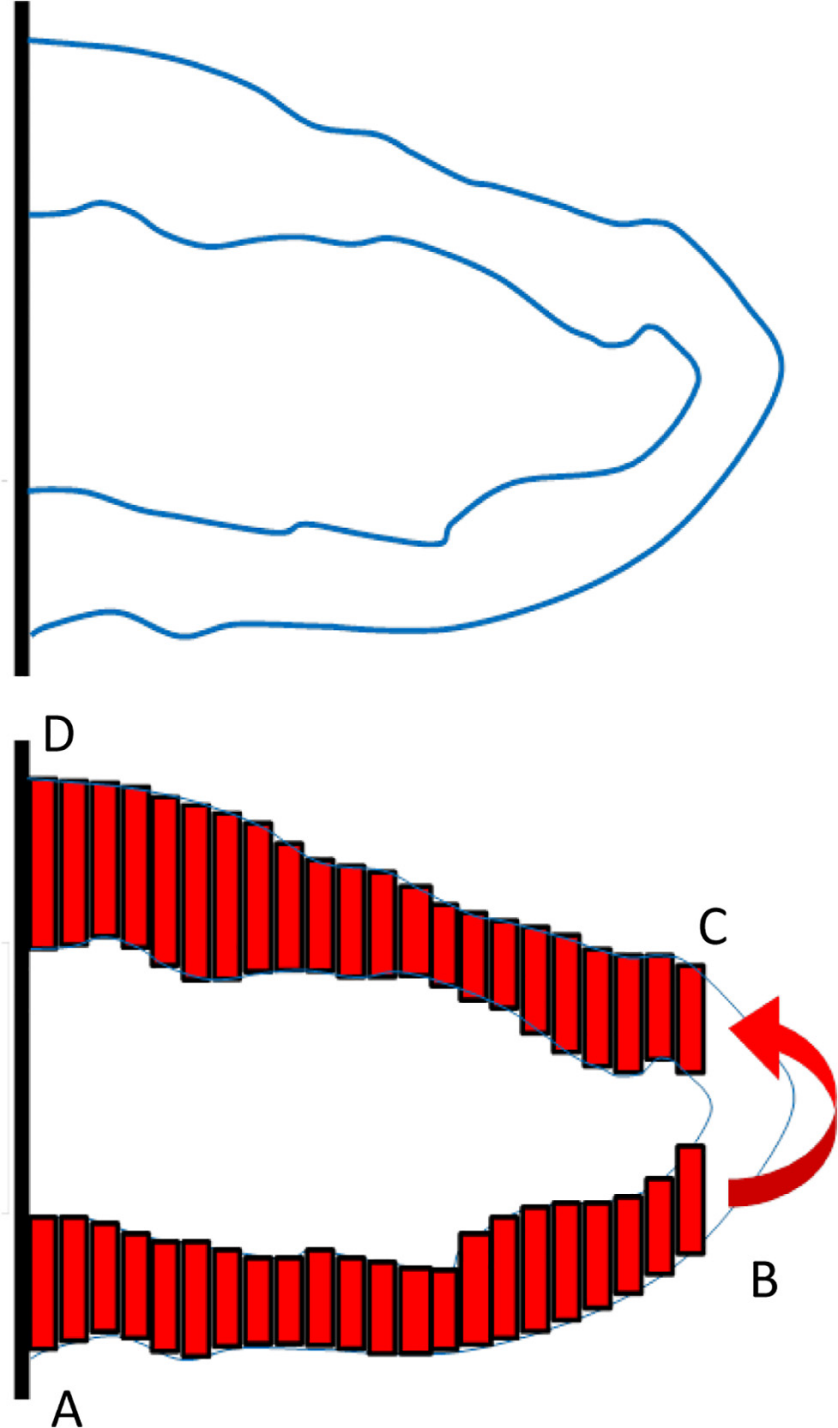
Schematic of the vessel segments assumed for flow calculations.

To implement the one-dimensional flow model in the vessels, it is assumed that the vessel consists of multiple one-dimensional segments. The segments width is one lattice site. The segments height is the vessel diameter that is different in each segment. The geometrical arrangement of a sample loop and the segments are shown in Fig 6.

The leakiness of the newly formed capillaries plays an important role in interstitial pressure distribution [66,70]; however, to our knowledge, interstitial pressure has no contribution in EC phenotype. Moreover, the rate of flow exchange due to vessel wall leakiness is negligible in comparison to axial flow in the capillaries [71,72]. Therefore, vessel wall leakiness in neglected in this model.

In the schematic presented in Fig 6, flow enters the loop from the parent vessel in point A, goes through the ABCD path, and turns back to the parent vessel in point D. The main reason for flow in the loop is the pressure difference in the parent vessel between points A and D. Though the parent vessel is an easier path for blood to flow from A to D, part of flow is bypassed in the loop. Since the effect of the turn in BC section cannot be observed in the one-dimensional model, it is assumed that the flow in point B enters directly to point C (shown by a curved arrow in Fig 6). For the few ECs in BC sections, average pressure in BC section is employed.

Considering the vessel segments as one-dimensional elements, the flow in each segment is calculated by:

$$Q=\frac{\pi }{128}\frac{\Delta P\;D^{4}}{L\mu }$$ (9)

where Δ*P* is the pressure difference in a segment, D is the segment height (vessel diameter), L is the segment width, and μ is blood viscosity.

**Fig 7:**
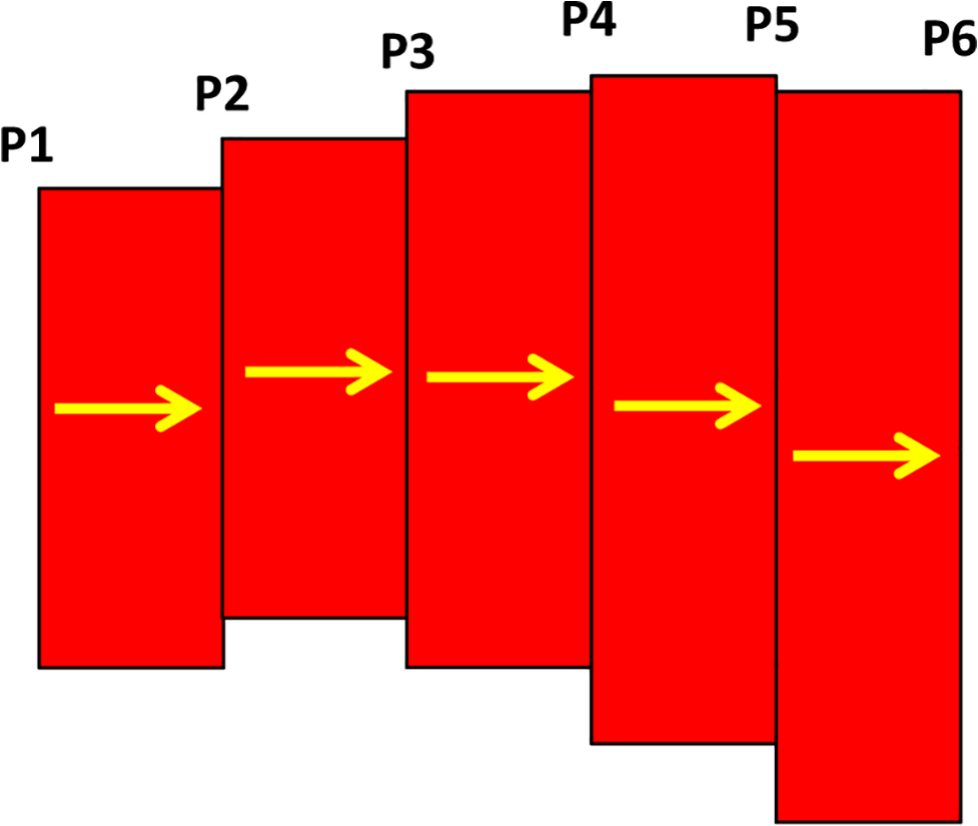
Few segments of a vessel from the loop.

Few segments of a vessel are shown in Fig 7. The pressure in each segment is shown by P_1_, P_2_, P_3_, …, P_n_, and the flow between segments is shown by yellow arrows. Mass conservation law results in equal flow in all segments.

$$Q=\frac{\pi }{128}\frac{P_{2}-P_{1}}{L\mu }D_{1}{}^{4}=\frac{\pi }{128}\frac{P_{3}-P_{2}}{L\mu }D_{2}{}^{4}=\frac{\pi }{128}\frac{P_{4}-P_{3}}{L\mu }D_{3}{}^{4}=\ldots$$ (10)

For each two adjacent segments, the equality of mass flow results in

$$\begin{array}{l} \left(P_{2}-P_{1}\right)D_{1}^{4}=\left(P_{3}-P_{2}\right)D_{2}^{4}\to P_{2}\left(D_{2}^{4}+D_{1}^{4}\right)-P_{1}D_{1}^{4}-P_{3}D_{2}^{4}=0\\ \left(P_{3}-P_{2}\right)D_{2}^{4}=\left(P_{4}-P_{3}\right)D_{3}^{4}\to P_{3}\left(D_{3}^{4}+D_{2}^{4}\right)-P_{2}D_{2}^{4}-P_{4}D_{3}^{4}=0 \end{array}$$ (11)

and for the last segment

$$\left(P_{n-1}-P_{n-2}\right)D_{n-2}^{4}=\left(P_{n}-P_{n-1}\right)D_{n-1}^{4}\to P_{n-1}\left(D_{n-1}^{4}+D_{n-2}^{4}\right)-P_{n-2}D_{n-2}^{4}-P_{n}D_{n-1}^{4}=0$$ (12)

where P_1_ and P_n_ are pressure in points A and D in Fig 6. The system of equation is solved simultaneously to obtain the pressure distribution in the loop.

$$\left[\begin{array}{cccccc} 1 & 0 & 0 & 0 & \ldots & 0\\ -D_{1}^{4} & D_{1}^{4}+D_{2}^{4} & -D_{2}^{4} & 0 & \ldots & 0\\ 0 & -D_{2}^{4} & D_{2}^{4}+D_{3}^{4} & -D_{3}^{4} & \ldots & 0\\ \vdots & \vdots & \vdots & \ddots & \vdots & \vdots \\ 0 & \ldots & 0 & -D_{n-2}^{4} & D_{n-1}^{4}+D_{n-2}^{4} & -D_{n-1}^{4}\\ 0 & 0 & 0 & \ldots & 0 & 1 \end{array}\right]\times \left[\begin{array}{c} P_{1}\\ P_{2}\\ P_{3}\\ \vdots \\ P_{n-1}\\ P_{n} \end{array}\right]=\left[\begin{array}{c} P_{A}\\ 0\\ 0\\ \vdots \\ 0\\ P_{D} \end{array}\right]$$ (13)

Having the pressure in each segment, the flow is calculated from Eq 10 and the wall shear stress is obtained subsequently as:

$$\tau_{w}=\frac{32Q\mu }{\pi D^{3}}$$ (14)

It should be mentioned that the flow is equal in all sections so once the flow is obtained for one section, it is not required to calculate the flow in other sections; however, obtaining the pressure difference in one section is not possible without solving the complete set of equations (Eq 13). The viscosity in Eq 14 depends on vessel diameter and blood hematocrit, which is discussed in the next section. With the value of shear stress in each segment, an average shear stress for each EC is calculated. The calculated values of shear stress in ECs are used to determine ECs phenotype alteration due to blood flow.

#### Blood Viscosity in Capillaries

The Poiseuille’s law is used for Newtonian fluid; however, blood has significant non-Newtonian properties at low Reynolds numbers. Blood viscosity in capillaries depends on the vessel diameter and hematocrit. To take advantage of Poiseuille’s law’s simplicity and use it to show the behavior of blood, it is helpful to define the apparent or effective blood viscosity.

$$\mu_{app}=\frac{\pi }{128}\frac{\Delta P\;D^{4}}{LQ}$$ (15)

Eq 15 is similar to Eq 9. Pries et al. [68,69] used data obtained from the results of 18 studies of human blood and combined the data with a parametric description of apparent blood viscosity relative to the plasma viscosity to define a mathematical function for apparent viscosity. They introduced this parameter as the relative apparent viscosity. A description of relative apparent viscosity as a function of tube diameter and hematocrit is as follows [68]:

$$\mu_{rel}=\left[1+\left(\mu_{45}-1\right)\frac{{\left(1-H\right)}^{C}}{{\left(1-0.45\right)}^{C}-1}{\left(\frac{D}{D-1.1}\right)}^{2}\right]{\left(\frac{D}{D-1.1}\right)}^{2}$$ (16)

μ_45_, the relative apparent blood viscosity for a fixed hematocrit of 0.45, is given by

$$\mu_{45}=6e^{-0.085D}+3.2-2.44\cdot e^{-0.06D^{0.645}}$$ (17)

where D is the vessel diameter (in mm) and C describes the shape of viscosity dependency on the hematocrit, defined as

$$C=\left(0.8+e^{-0.075D}\right)\left(-1+\frac{1}{1+10^{-11}D^{12}}\right)+\frac{1}{1+10^{-11}D^{12}}$$ (18)

The apparent blood viscosity is defined as

$$\mu_{app}=\mu_{plasma}\times \mu_{rel}$$

In this article, the hematocrit is assumed to be 0.45. For each segment of the vessel, the apparent blood viscosity is calculated, and then it is used in Eqs 9 and 14 for calculation of blood flow and shear stress.

### Solution algorithm

The cellular scale of this model is the body of the model, to draw an analogy, while the intracellular scale is its brain. At the tissue scale, ECs receive signals from the environment and, in the case of blood flow, from shear stress induced by blood flow. Then, these signals are processed and decisions are made at the intracellular scale. The cellular scale is the level at which the results of the cell decision making machinery are applied and the changes are observed.

As mentioned before, at the cellular scale, the system energy controls the solution. The total system energy or the Hamiltonian is the sum of the energy terms for adhesion, growth, chemotaxis, continuity, and anastomosis.

$$\begin{array}{l} E_{total}=E_{adhesion}+E_{growth\;constr.}+E_{chemotaxix}+E_{continuity}+E_{tip\;cell\;attrac.}\\ =\sum_{sites}J_{\tau ,\tau \prime }\left(1-\delta_{\sigma ,\sigma \prime }\right)+{\sum_{cells}\gamma_{t}\left(a_{\sigma }-A_{\sigma }^{T}\right)}^{2}+\sum_{sites}\chi_{\sigma }\Delta V+\sum_{cells}\alpha \left(1-\delta_{a_{\sigma },a_{\sigma }^{\prime }}\right)+{\sum_{tip\;cells}\psi \left({\bar{X}}_{1}-{\bar{X}}_{2}\right)}^{2} \end{array}$$ (19)

The connection between the intracellular and cellular scales is established through Eq 19. Based on the EC phenotype predicted from intracellular scale, migration and proliferation of ECs is applied to the model. If proliferation exist in the cell phenotype, the cell is able to grow, else the term related to growth in Eq 19 is not calculated. Moreover, based on the predicted phenotype for migration, different values for chemotactic sensitivity is used in the model. In other words, the intracellular scale regulates application of Eq 19 to the model. Table 2 shows the values of the parameters used in the model. The applicable range of parameters in the model and a sensitivity analysis on the parameters for the model of single sprout is reported by Bauer et al. [12].

**Table 2:** Parameters used in the model and corresponding references.

Parameter	Symbol	Value	Ref.
VEGF Diffusion Equation Parameters			
VEGF Diffusion	D	3.6 x 10 ^−4^ cm^2^/h	[73]
VEGF Decay	*λ*	0.9375 h^-1^	[73]
VEGF Uptake	*β*	0.06 pg/EC/hr	[74]
VEGF Source	S	0.035 pg/pixel	[75]
Activation Threshold	*v*_*a*_	0.0001 pg	[1]
Cellular Pott’s Model Parameters			
EC–EC Adhesion	*J*_*ee*_	30 E/L	[12]
EC–Fluid Adhesion	*J*_*ef*_	76 E/L	[12]
EC–Matrix Adhesion	*J*_*em*_	66 E/L	[12]
Fluid–Fluid Adhesion	*J*_*ff*_	71 E/L	[12]
Fluid–Matrix Adhesion	*J*_*fm*_	85 E/L	[12]
Matrix–Matrix Adhesion	*J*_*mm*_	85 E/L	[12]
EC Membrane Elasticity	*γ*_*e*_	0.8 E/L^4^	[12]
M&P Cell Chemotactic Sensitivity	*χ*_*MP*_	-1.55x1.11x10^6^ E/conc	[12]
M Cell Chemotactic Sensitivity	*χ*_*M*_	-1.42x1.11x10^6^ E/conc	[12]
P Cell Chemotactic Sensitivity	*χ*_*P*_	-1.40x1.11x10^6^ E/conc	[12]
Intracellular Continuity	*α*	300 E/L	[12]
Boltzmann Temperature	kT	2.5 E	[12]
Attraction Coefficient	*ψ*	1600 E/L^2^	
Blood Flow Parameters			
Blood Hematocrit	H	0.45	[76]
Plasma Viscosity	μ_plasma_	0.0012 Pa.s	[77]
Shear Stress Activation Threshold	τ_threshold_	0.2 dyne/cm2	[67]

The units of the parameters in Table 2 are in accordance with Eq 19, e.g. E represents the energy and L is the length scale in the model.

Cellular Pott’s model changes the system state based on probability. As shown in Fig 5, each lattice site has an assigned number that specifies its entity. A lattice site with the assigned number *σ* is selected randomly. The total system energy is calculated. One of the unlikely neighbors of the lattice site is chosen randomly. The assigned number of the lattice site is replaced by its unlikely neighbor. The total system energy is calculated after system change. If the total system energy is reduced due to this change, it is accepted; otherwise, the update is accepted with a Boltzmann probability:

$$P_{accept.}=\exp\left(\frac{-\Delta E}{kT}\right)\;\Delta E>0$$ (20)

where k is the Boltzmann constant and T is a measure of system disorder.

If the domain has NxM lattice sites, this process is repeated NxM times to go through one Monte Carlo step (MCS). MCS is the temporal scale of the model, which in this model is approximately 1 minute [12,13]. The time scale of this model is obtained by equating the required time for cell division in the model with EC cycle duration [12].

The time scales in the different scales of the model are different. Comparing with cellular scale, the time scale of tissue and molecular scales are much smaller. Since the cellular scale events drive the model, the tissue and molecular scales events also conform with cellular scale and therefore the molecular and tissue scales events are calculated once in each MCS.

## Results and Discussion

The solution Domain is divided into 240x480 lattice sites. The tumor is assumed as a linear tumor on the right side and the parent vessel on the left side of the domain. Two sprouts are assumed to start migration from the parent vessel.

### Closed loop formation

**Fig 8:**
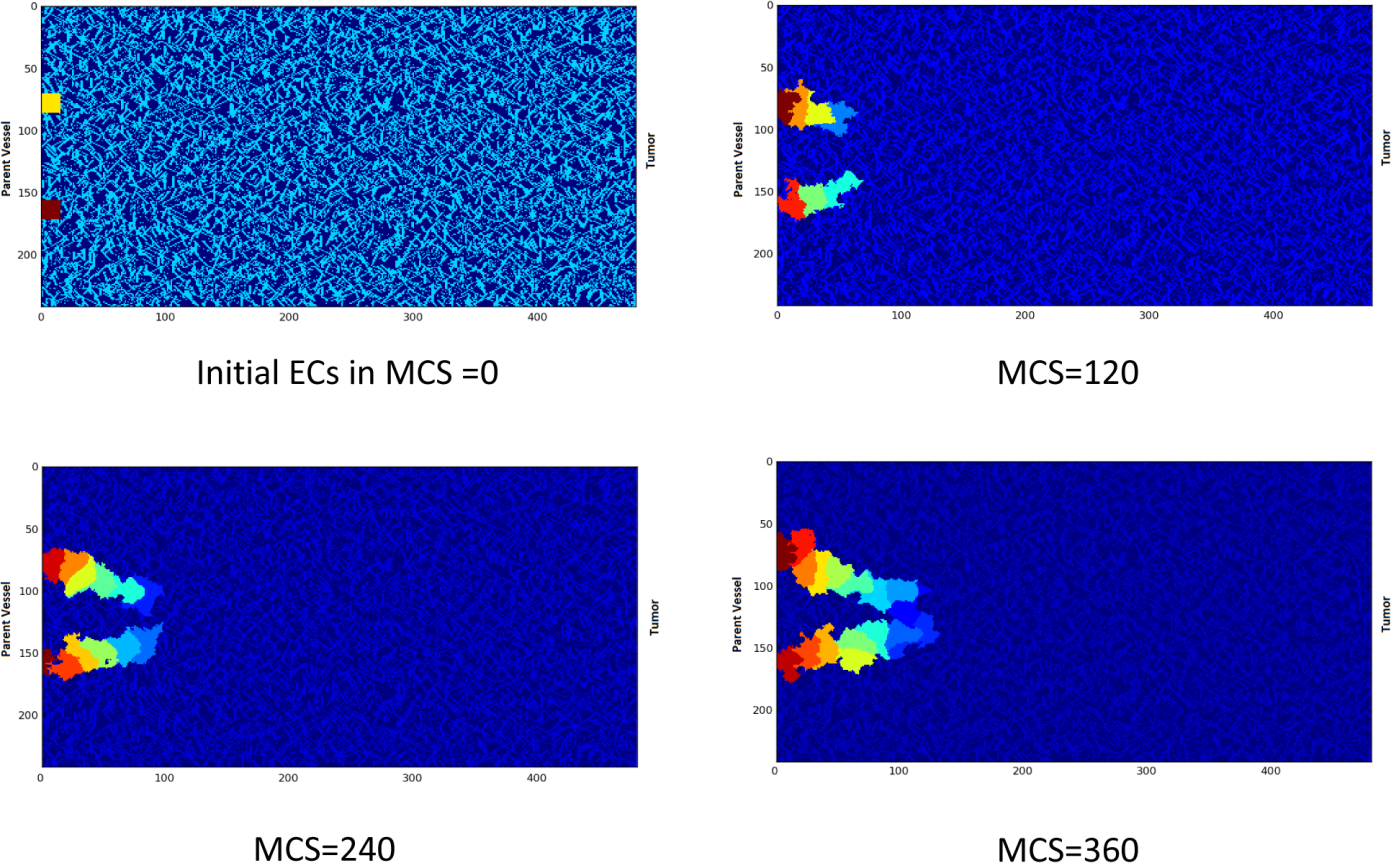
Closed loop formation in the model. The X and Y-axis show the domain size in μm.

A closed loop is formed by anastomosis of two sprouts. The anastomosis is possible to begin at any time during angiogenesis, and different loop sizes are feasible. The parameter ψ in the Hamiltonian controls the initial size of the loop. After formation of a closed loop, blood flow starts in the loop. This is the key event that survives the loop. The focus of the current study is on the loop condition after blood flow starts. To compare the loop evolution in different conditions, a single closed loop is obtained once and is used as the starting point of the model in different conditions, i.e. the model does not start from two single ECs every time, rather the model starts from the closed loop every time. The closed loop formation steps are shown in Fig 8.

### Cell phenotype alteration due to flow in the loop

When blood flows in the closed loop, it acts as the main regulator of cell phenotype. ECs are activated by shear stress magnitudes as low as 0.2 dyne/cm^2^ [64,67]. Activation means that both integrin and RTK are activated and this is not dependent to their specific ligands [24,43]; however, activation of RTK and integrin depends on activation of VE-cadherin [23].

**Fig 9:**
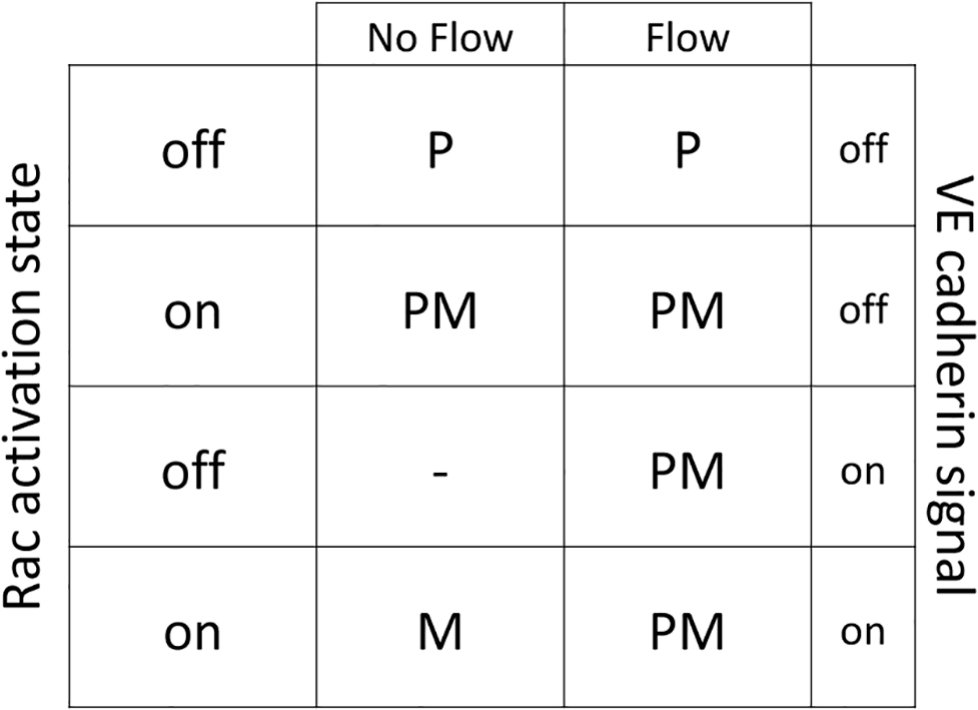
EC phenotype considering both flow and no flow conditions. The outputs are determined by letters, P: Proliferation, M: Migration, PM: Proliferation and Migration.

In cell phenotype determination before loop formation, apoptosis is dominant cell phenotype except when both RTK and integrin signals are active [16,17]. Similarly, flow activates both RTK and integrin; as such, a hybrid map is derived for EC phenotype with and without flow. The hybrid map is shown in Fig 9. In determining cell phenotype from Fig 9, it is assumed that both integrin and RTK are active; else, the apoptotic signal is activated in ECs. Activation of VE-cadherin is related to cell-cell contacts. Rac activation state also plays a major role in cell phenotype determination (see [17], for more explanation about the role of Rac and VE-cadherin in the ECs signaling cascade in angiogenesis).

When blood flows in the loop and creates shear stress on capillary walls, activation of integrin and RTK depends on VE-cadherin. This means that when VE-cadherin is not activated, the results are similar to no flow conditions. By contrast, when the VE-cadherin signal is active, the EC starts to proliferate and migrate, without relation to the Rac state.

### Loop collapses when flow phenotype alteration is neglected

**Fig 10:**
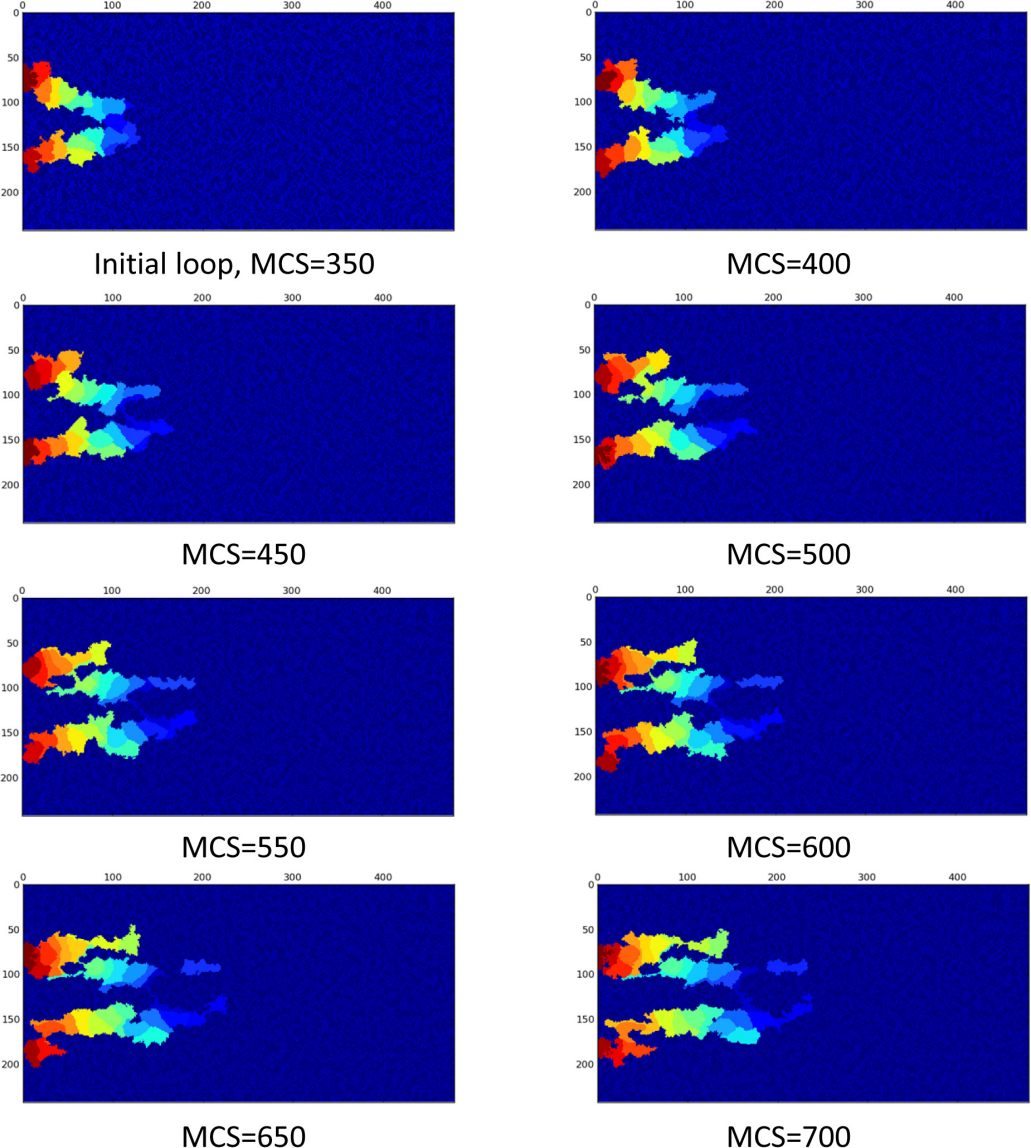
Loop collapses when effect of flow in the cell phenotype is neglected. The complete collapse is obvious after 700 MCS. The X and Y-axis show the domain size in μm.

Loop formation is the starting point of important changes in the capillaries. The capillaries are not always survived, i.e. the collapse is possible. Blood flow and the resulting shear stress in capillaries have a pivotal role in maintaining homoeostasis of vessels [23]. In numerical models of sprouting angiogenesis, when flow is involved in the model, there is usually a criterion for capillaries to remain in the capillary network, which is a minimum amount of flow; otherwise the capillary will be pruned [10,65]. The model developed in the current study incorporates and studies the effect of flow in loop survival, elongation, and stabilization. To show what happens for a loop when blood flow is absent, a loop without flow is modeled, i.e., after closed loop formation, no flow is considered in the loop. In this case, the EC phenotypes are determined without blood flow consideration. Results are shown in Fig 10.

Fig 10 shows that, when blood flow effect is not considered in the cell phenotype, the loop loses its integrity and collapses.

### Loop elongates when flow alters EC phenotype

**Fig 11:**
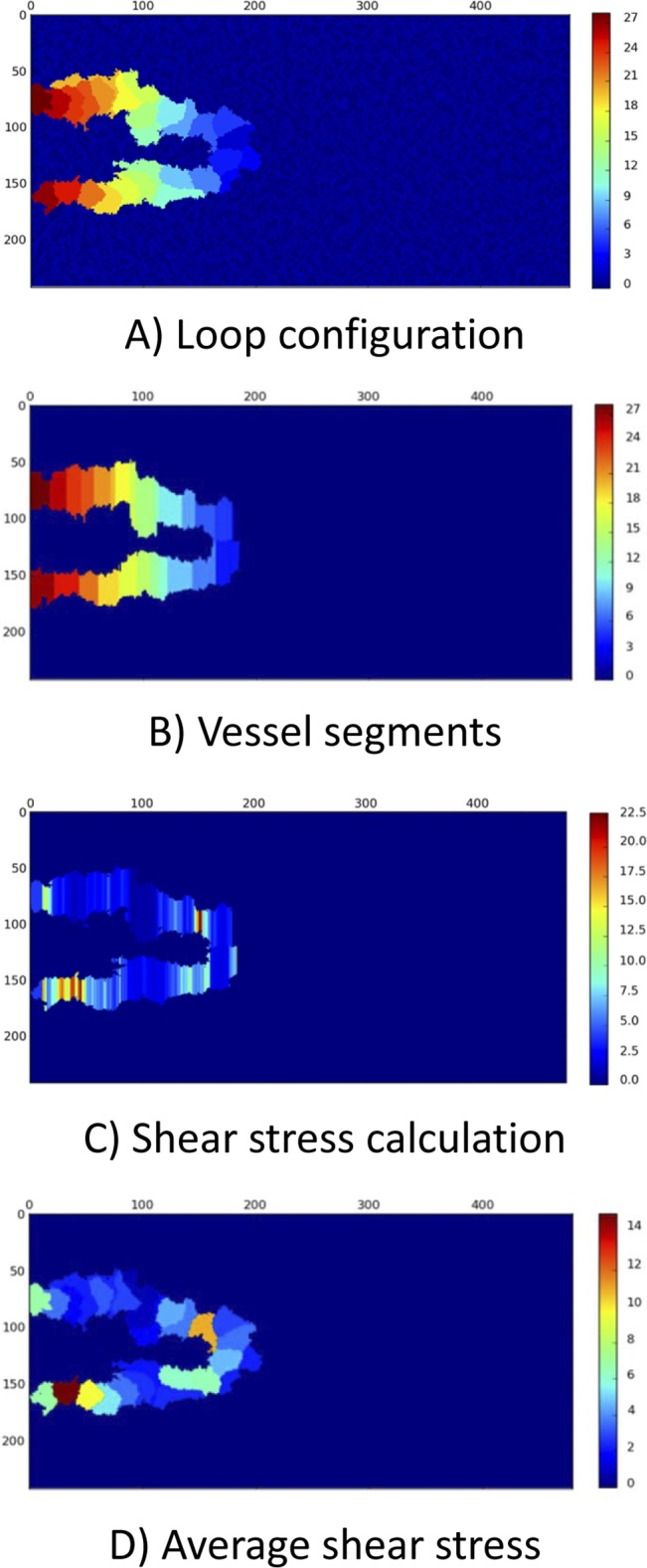
The process of shear stress calculation in the loop. The X and Y-axis show the domain size in μm. A) The configuration of the loop changes in each MCS. The loop configuration is the input to the flow calculation procedure. The color bar shows the EC identity in the solution. B) The current configuration of loop is approximated by vessel segments. Some geometrical simplifications may be required. The color bar is identity of ECs in the solution. Based on the portion of each EC in segments, each segment is assigned to specific EC. C) The pressure, flow, and shear stress are calculated in each segment. The color bar shows shear stress in dyne/cm2. In areas with lower segment diameter, shear stress is higher. D) The average shear stress on each EC is calculated and used for shear stress activation of ECs. The color bar shows the average shear stress in dyne/cm2.

When blood flows in the loop, the main regulator of EC phenotype is the shear stress induced by blood flow. In each MCS, the loop is approximated by the vessel segments, as shown in Fig 6. Then, flow, pressure, and shear stress are calculated in each segment. Considering the portion of ECs in each segment, an average value for shear stress is calculated for each EC. In this model it is assumed that if the average shear stress in the EC is beyond a specified threshold, it activates the signaling cascade of flow; otherwise, the EC phenotype is determined from no flow condition. The calculation procedure for a sample configuration of ECs in the loop is shown in Fig 11.

**Fig 12:**
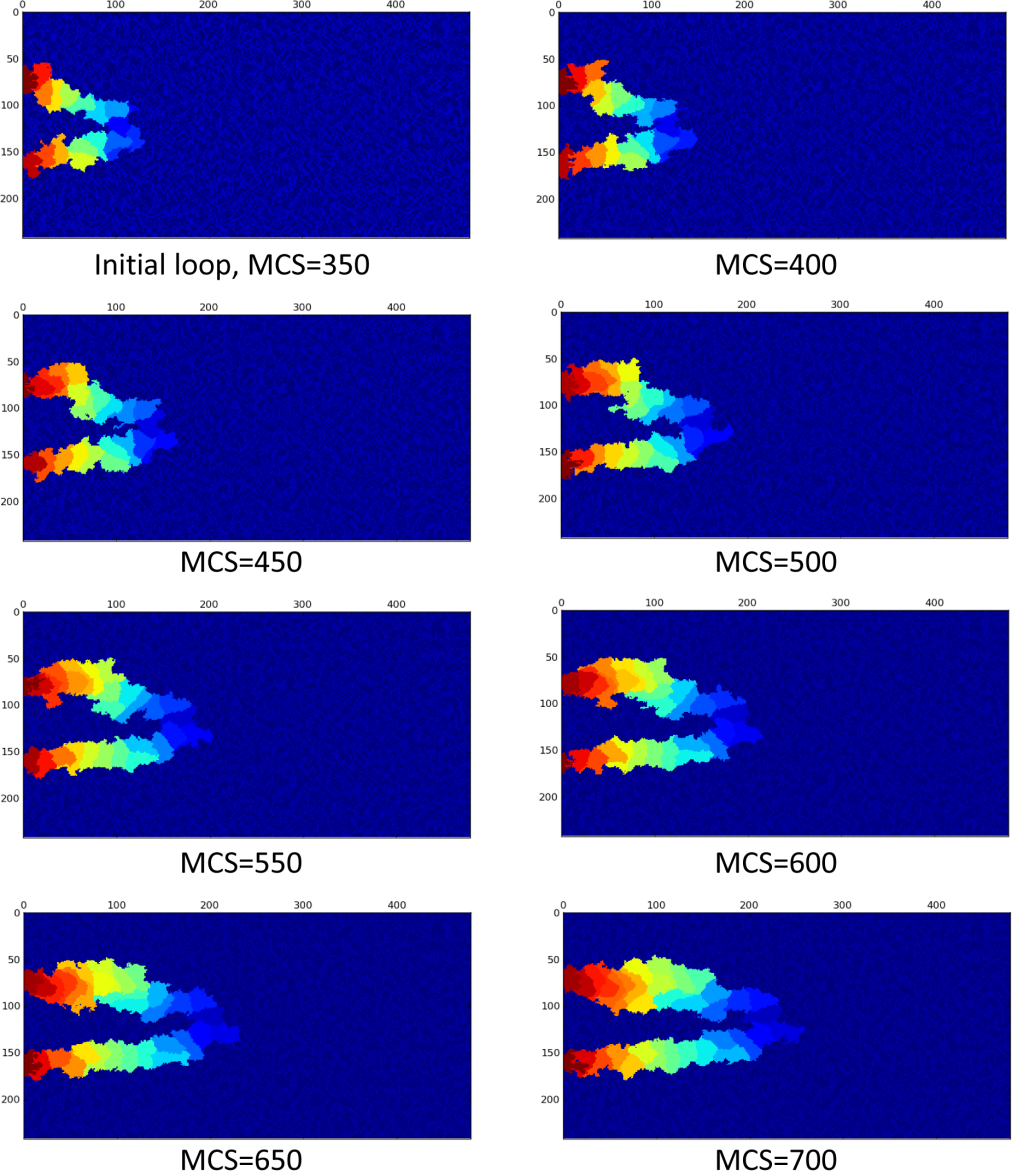
Survival of loop when both proliferation and migration signals are activated by flow in the loop. EC color is representative of their identity in the solution. The X and Y-axis show the domain size in μm.

Applying the blood flow in the model and consequent cell phenotype alteration changes the loop configuration drastically. Assuming 30 Pa Pressure difference between loop inlet and outlet, Fig 12 shows a loop in which blood flow induces shear stress and consequently, ECs alter their phenotype according to the map presented in Fig 9.

#### Effect of blood viscosity

**Fig 13:**
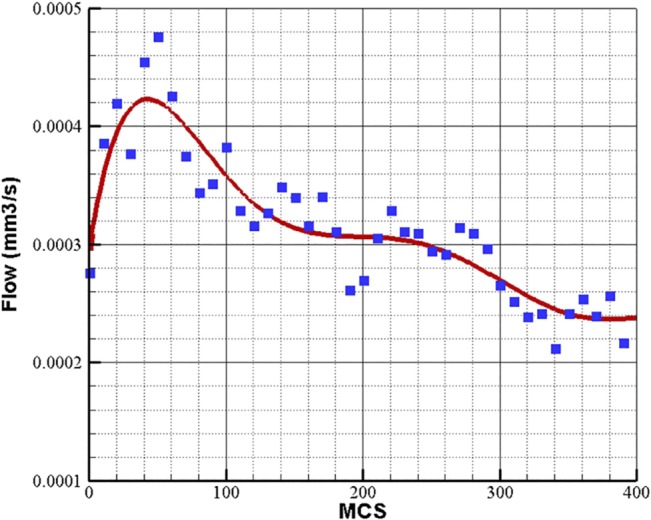
Blood flow in a closed loop with 30 Pa pressure difference in inlet and outlet of the loop. Blood viscosity is assumed constant 0.0035 Pa.s. The flow data is averaged over 10 independent simulations.

Assuming 30 Pa pressure difference between inlet and outlet of the loop, the flow is calculated and plotted in Fig 13 for constant blood viscosity of 0.0035 Pa.s. Due to stochastic nature of the methods used in the simulations, different flow data is obtained in each run. To present variation of flow during loop elongation in a single diagram, the flow data is averaged over 10 independent simulations.

**Fig 14:**
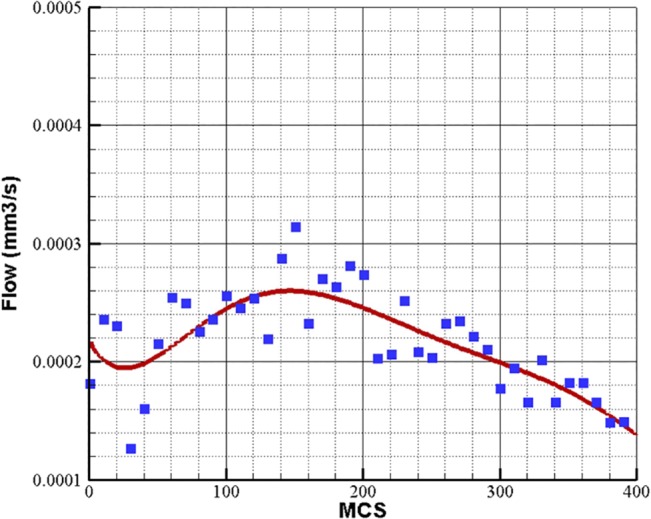
Blood flow in a closed loop with 30 Pa pressure difference in inlet and outlet of the loop. Blood viscosity depends on vessel diameter and hematocrit. The flow data is averaged over 10 independent simulations.

Considering the viscosity dependency on blood hematocrit and vessel diameter results in different flow quantity in the loop. Using this assumption, the average flow in the loop with 30 Pa pressure difference between inlet and outlet of the loop is shown in Fig 14.

#### Effect of pressure difference

Blood flow in the loop depends directly on the pressure difference in the loop inlet and outlet. The physiological value of pressure difference in a parent vessel is reported to be 20 mmHg for 2 cm of vessel [78]. The variation of pressure along the parent vessel can be assumed linear [76], so the pressure variation along the parent vessel is 0.001 mmHg/μm. In the current study, the distance between the loop inlet and outlet is variable during solution; however, its mean value is around 100 μm. The pressure difference between the loop inlet and outlet is obtained 0.1 mmHg or 13.3 Pa. In this work, the range of pressure difference values is considered to vary between 5 and 30 Pa.

**Fig 15:**
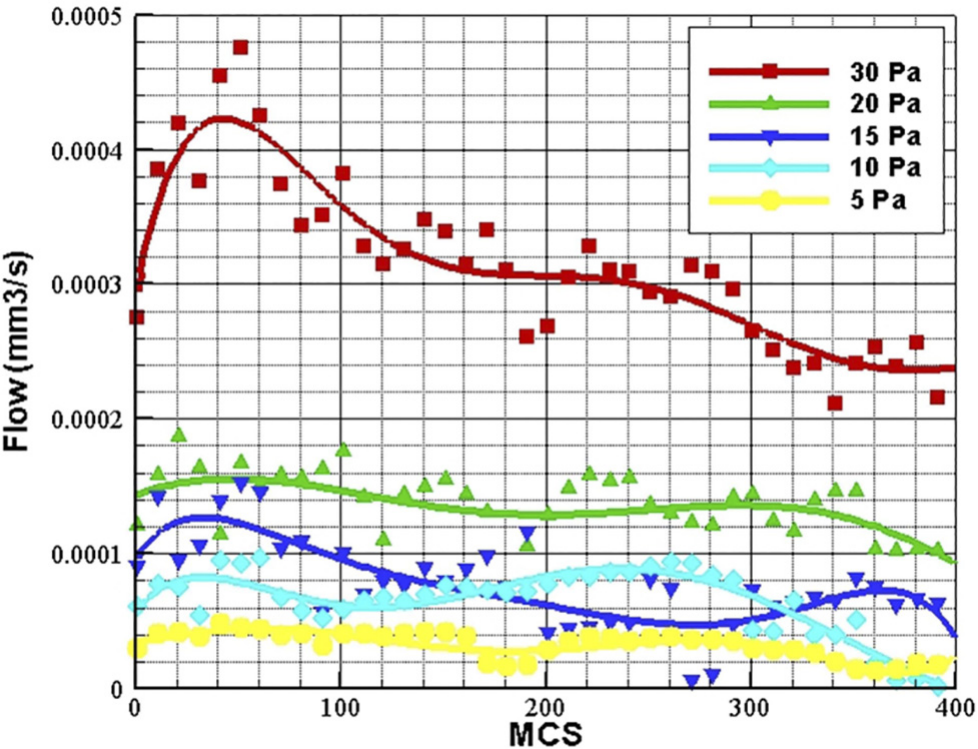
Flow variations in different values of pressure difference during loop elongation. The flow data is averaged over 10 independent simulations.

Pressure difference between the two sprouts’ connection to parent vessel is the source of blood flow in the loop. The flow quantity and consequently the shear stress directly depend on the pressure difference. It can be concluded that, depending on the solution domain geometry and vessel size, there is a threshold for pressure difference, in which the flow in the loop is not enough to induce shear stress and alter ECs phenotype. To investigate this issue, loops with different pressure difference in inlet and outlet are tested and results are presented in Fig 15.

As observed in Fig 15, flow is increased as pressure difference is increased. The loop is not able to keep its structure in pressure difference lower than 3 Pa. In pressure difference of 4 Pa and higher, the loop development in normal. The main reason that the loop cannot develop normally in low pressure differences, is low flow and consequently, low shear stress, which cannot induce ECs to alter their phenotype. The flow depends directly on pressure difference in the loop; however, during the solution some discrepancies may be observed. The flow quantity in 15 Pa pressure difference is higher than 10 Pa pressure difference; however, as shown in Fig 15, the loop with 10 Pa pressure difference has higher flow quantity than 15 Pa between MCS 200 and 300. This is due to stochastic nature of the model geometry and dependency of flow to other parameters like vessel diameter. However, the general trend of flow shows higher flow quantity in 15 Pa.

#### Phenotype analysis in a loop

**Fig 16:**
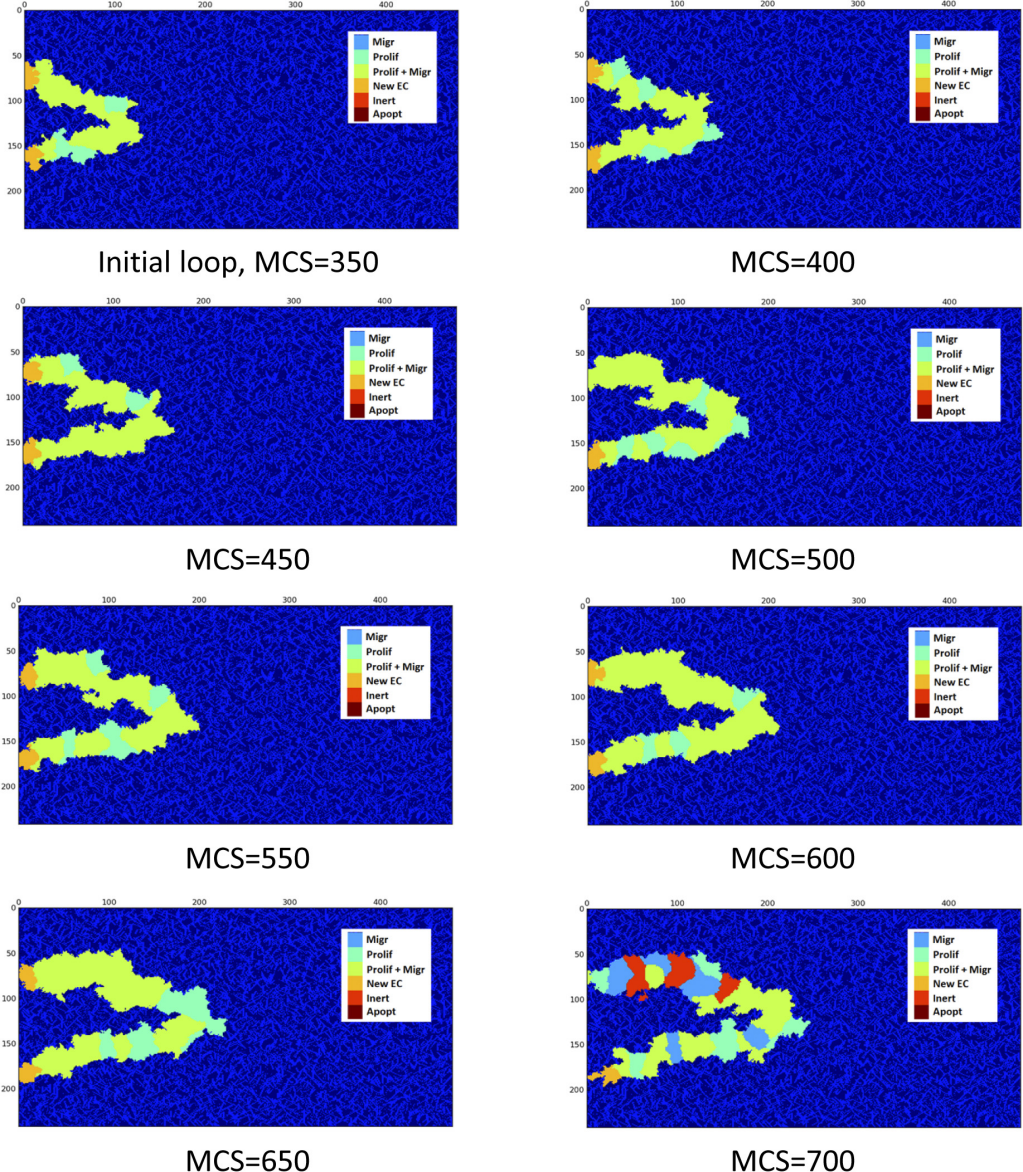
Phenotype distribution in ECs of a survived loop. Proliferation and migration is the dominant phenotype.

In order to analyze the role of EC phenotype in loop development, the phenotype distribution of ECs during the loop elongation in a survived loop is shown in Fig 16.

The main part of the model that enables it to predict loop elongation is the phenotype determination map. In fact, based on the received signals from the environment, proper choice of the phenotype is the key to capture real physics of the phenomenon.

**Fig 17:**
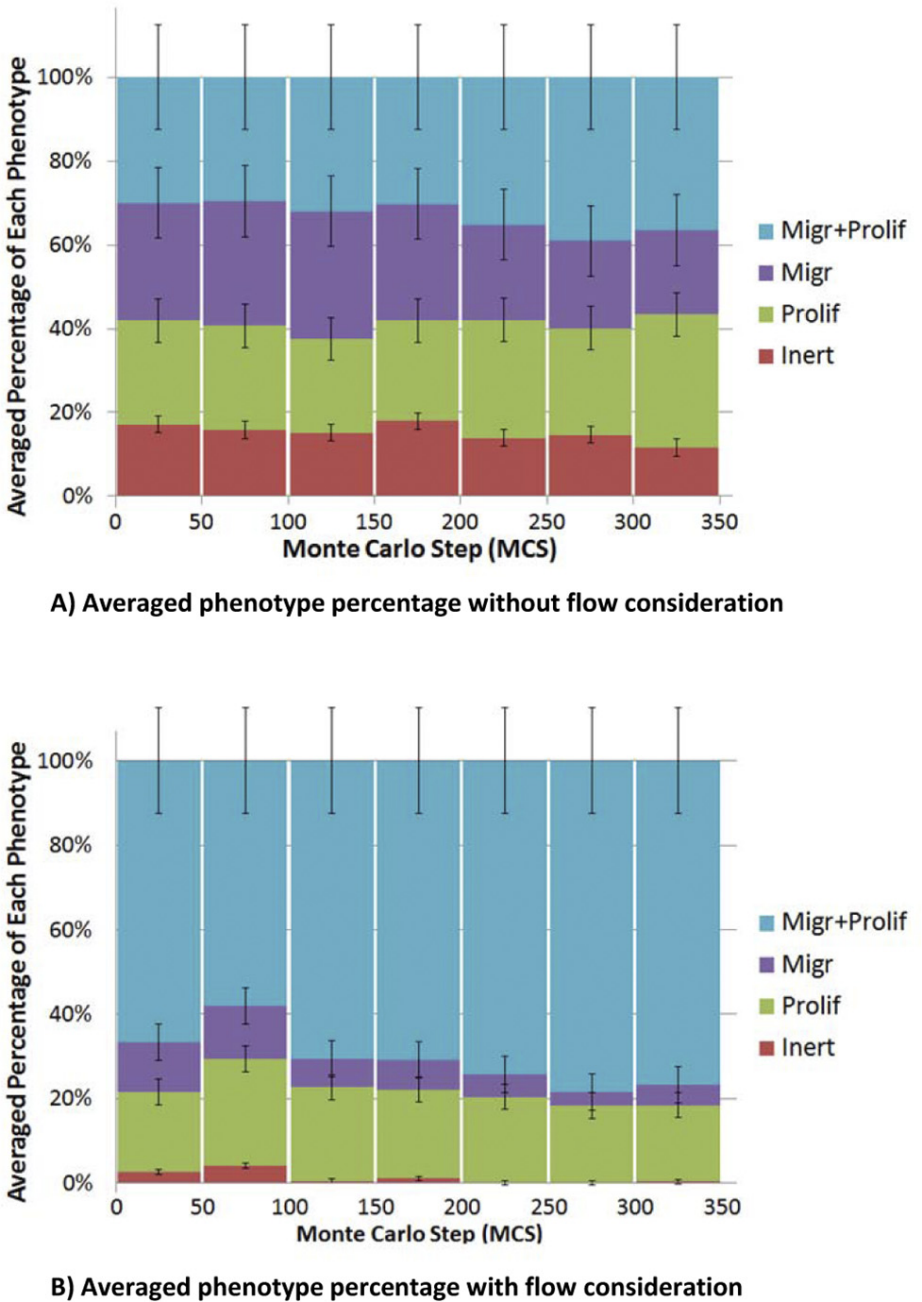
Stacked area diagram for portion of each phenotype in the loop during the solution. A) The case without flow which leads to loop collapse. B) The case with flow which leads to loop survival.

Comparison of Fig 10 and Fig 12 clearly shows the effect of blood flow in a loop. In Fig 10, the loop loses its integrity during its growth, while the structure of the closed loop is maintained during loop elongation in Fig 12. The only difference in these results is consideration of cell phenotype alteration due to blood flow. This issue shows that suitable phenotype activation is the key in loop survival. Fig 16 shows that proliferation and migration are the dominant phenotypes during loop elongation. To assess the phenotype distribution in a loop, stacked area diagrams for portion of each phenotype during the solution is shown in Fig 17. Each color in the bars in Fig 17 shows the average portion of each phenotype during 50 MCS. Similar to the flow data in Fig 13 to Fig 15, the phenotype data is averaged over 10 independent simulations.

Fig 17A shows the averaged phenotype percentage for a loop without blood flow. Fig 17A shows that 14–18% of ECs are inert during solution. By contrast, Fig 17B shows the averaged phenotype percentage for a loop with blood flow, and it is seen that the inert signal is less than 5%. This issue shows the key role of proliferation and migration in loop elongation and integrity. Moreover, migration and proliferation phenotype in Fig 17A has a higher portion in comparison to migration or proliferation phenotypes, but no specific phenotype can be assumed as the dominant phenotype. By contrast, in Fig 17B, the dominant phenotype is proliferation and migration. Comparison of Fig 17A and 17B, clearly reveals the reason behind the loop survival.

#### Effect of flow on vessel diameter

**Fig 18:**
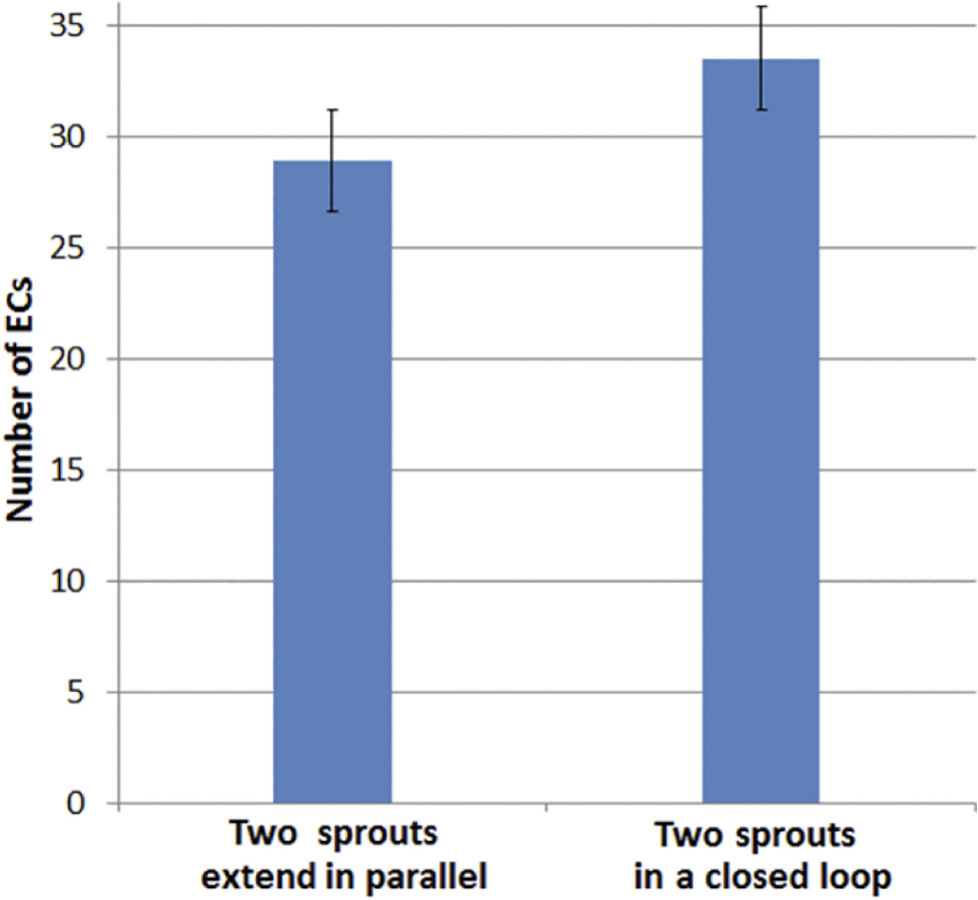
Comparison of number of ECs between two sprouts extend in parallel and two sprouts in a closed loop. The number of ECs is averaged over 10 independent simulations.

In addition to loop survival, flow changes the diameter of capillaries [79]. As a qualitative comparison, Fig 18 shows the number of ECs in a loop and two sprouts that extend in parallel. The number of ECs is averaged for 10 independent simulations with identical initial conditions. The criterion to stop the simulation is 230 *μm* extension of one of the parallel sprouts or the loop in the domain.

As the number of ECs in Fig 18, indicates, the loop has more ECs in comparison to the single sprouts extend in parallel. This means higher vessel diameter in the loop, which is naturally induced by blood flow.

### Model validation

Loop elongation is a phenomenon that is observed in the initial stages of sprouting angiogenesis. Several *in vivo* experimental models have reported loop formation and elongation [2,3]. A key point in the present work is that the prediction of loop elongation and maintaining blood vessels integrity was not imposed within the model *a priori*, i.e. it is the result of the model, which is obtained by combining molecular, cellular, and tissue scale dynamics.

One of the earliest studies on the effect of blood flow on the vessels is performed by Clark [80]. Early studies by Clark showed that low values of shear stress (decrease or halt in blood flow) in small vessels results in vessel regression, where high values of blood flow induced shear stress stabilizes the vessels. In a more recent study, Meeson et al. studied the effect of flow on the capillary regression [81]. In this model, combining vital and histological analysis, the fate of capillary segments following cessation of flow is investigated. The results show that, after an initiating apoptosis in the upstream, a block to the flow due to lumen restriction occurs. The block in the flow induces apoptosis in the downstream ECs, which ultimately results in capillary regression. This study reveals that, even simple structures like a capillary segment are not able to survive without flow. The major role of shear stress in vessel regression is also demonstrated by Pries et al. [82]. In this study, combining experimental and mathematical models, Pries et al. obtained a relation between vessel diameter and wall shear stress. A direct linear like relation is obtained between shear stress and vessel diameter, which clearly shows regression of vessel in low shear stress. Taken together, it is concluded that vessel formation and maintenance strongly depends on fluid shear stress [83]. Accordingly, the model presented in the current study clearly shows the vessel formation and stabilization due to fluid shear stress. The general results of the model agree with experiments; however, quantitative validation is also investigated.

The problem investigated in this work consists of two steps. The first step is loop formation, which single sprouts extend and the phenotype is determined based on the no flow condition. The second step is loop development, which uses the phenotypes obtained with flow. Similarly, quantitative validation of the model also consists of two parts. The first part is the sprout extension before loop formation, and the second part is loop elongation after loop formation.

Sprout extension speed in angiogenesis is measured by Kearney et al. [84]. In this study, primitive vessels are formed by *in vitro* differentiation of embryonic stem cells. In a time period of 10 hours with 1 minute time interval, the average extension speed of new sprouts is measured at a rate of 14 *μm* / *hr*. The range of extension speed is measured from 5 to 27 *μm* / *hr* [84].

For loop elongation, the main *in vivo* experimental model for quantitative validation is the rabbit cornea assay [2,85]. Gimbrone et al. built an experimental model for tumor growth and neovascularization using the rabbit cornea [2]. This work studies the host angiogenic response to tumor cells and TAFs. The interesting part of this work is the host neovascular response, in which loop formation and its growth as elongated hairpins is reported. The initial loop extension speed is estimated at the rate of 0.5 mm/day. The ocular micrometer measurement error is 0.1mm and therefore the extension speed is estimated 20.8±4.2*μm* / *hr*.

**Fig 19:**
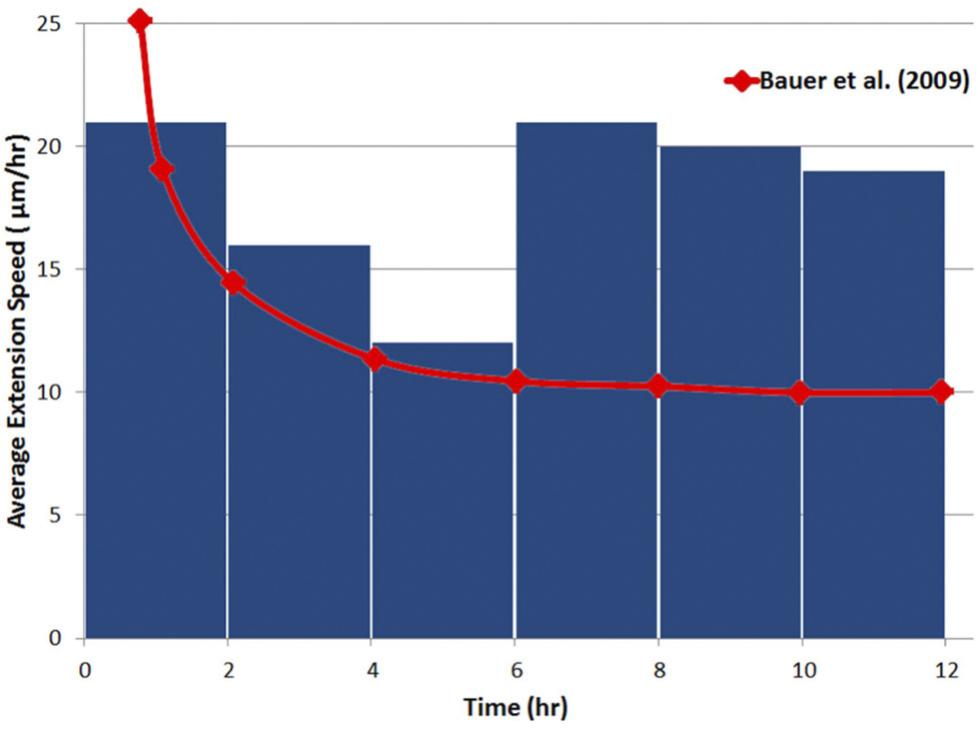
Extension speed of sprouts, averaged in time intervals of 2 hr. After 6hr, a loop is formed and a jump in extension speed occurs.

The reported extension speeds in experimental data show different values for single sprouts and loops. Though the difference may be interpreted as a common statistical difference in measurements and the experimental conditions, our results propose a different reason. Fig 19 shows the calculated average extension speed of sprouts that are averaged over time intervals of 2hr. The jump in extension speed is coincident with loop formation. The red curve shows the extension speed in the model of Bauer et al. [12] at a matrix density of 0.4. The model in [12] does not include loop formation, and there is excellent agreement between the extension speeds before loop formation. The current model predicts extension speeds that reduce as the sprouts extends; however, loop formation makes a jump in the extension speed.

Table 3 shows averaged extension speeds of the proposed model in this study and the measured extension speeds of the *in vivo* and *in vitro* experiments. The agreement of the model with experimental results is seen in Table 3.

**Table 3:** Average extension speeds of single sprouts and loops.

	Experiment	Model	Experiment Ref.
Single Sprouts	14 *μm*/*hr* (10 hr ave.)	16.3 *μm*/*hr* (6 hr ave.)	[84]
Loop	20.8 ± 4.2 *μm/hr*	20 *μm*/*hr*	[2]
