# Correction: The Vital Role of Blood Flow-Induced Proliferation and Migration in Capillary Network Formation in a Multiscale Model of Angiogenesis

**DOI:** 10.1371/journal.pone.0175601

**Published:** 2017-04-06

**Authors:** Hossein Bazmara, Madjid Soltani, Mostafa Sefidgar, Majid Bazargan, Mojtaba Mousavi Naeenian, Arman Rahmim

A reference is omitted from the fourth sentence of the last paragraph in the Introduction. The sentence should read: In addition, the heterogeneous ECM structure is considered at the cellular scale (Bauer, 2007).

The reference is: Bauer AL, A Multi-Scale Cell-Based Model to Simulate and Elucidate the Mechanisms Controlling Tumor-Induced Angiogenesis. Ph.D. Thesis, The University of Michigan. 2007.

Two references are omitted from the second sentence of the second paragraph under the subheading “Growth and migration of ECs” in the Materials and Methods section. The sentence should read: The adhesion energy is computed as follows by [12,13]

$$E_{adhesion}=\sum_{sites}J_{\tau ,\tau^{\prime }}\left(1-\delta_{\sigma ,\sigma^{\prime }}\right)$$ (1)

A reference is omitted from the fifth sentence of the fourth paragraph under the subheading “Growth and migration of ECs” in the Materials and Methods section. The sentence should read: The chemotactic contribution is [12]

$$E_{Chemotaxis}=\sum_{sites}\chi_{\sigma }\Delta V$$ (3)

where χ_σ_ is the effective chemical potential and ΔV is VEGF gradient.

Two references are omitted from the sixth sentence of the first paragraph under the subheading “Diffusion of TAFs in ECM” in the Materials and Methods section. The sentence should read: VEGF distribution in the domain is governed by a partial differential equation, which considers diffusion, decay, and uptake of VEGF [12]:

$$\frac{\partial V}{\partial t}=D\nabla^{2}V-\lambda V-B(x,y,V)$$ (6)

where V is VEGF concentration, D is diffusion coefficient of VEGF, λ is decay rate of VEGF, and *B* is a function expressing VEGF binding to VEGFR2 [12, 13].

Two references are omitted from the third sentence of the second paragraph under the subheading “Diffusion of TAFs in ECM” in the Materials and Methods section. The sentence should read: Assuming a rectangular domain, with a linear tumor in one side and a vessel in the opposite side, the boundary and initial conditions are imposed [12,13].

An acknowledgement was omitted from the article. The authors would like to show their gratitude to Amy L. Bauer for sharing the ANGIO 2D (Bauer et al., 2007), a python-based code for generating the ECM.

The reference is: Bauer AL, A Multi-Scale Cell-Based Model to Simulate and Elucidate the Mechanisms Controlling Tumor-Induced Angiogenesis. Ph.D. Thesis, The University of Michigan. 2007.
